# *Giardia lamblia* Decreases NF-κB p65^RelA^ Protein Levels and Modulates LPS-Induced Pro-Inflammatory Response in Macrophages

**DOI:** 10.1038/s41598-020-63231-0

**Published:** 2020-04-10

**Authors:** Clarissa Perez Faria, Bruno Miguel Neves, Ágata Lourenço, Maria Teresa Cruz, João D. Martins, Ana Silva, Sónia Pereira, Maria do Céu Sousa

**Affiliations:** 1 Faculty of Pharmacy, University of Coimbra, Coimbra, Portugal; 2 Center for Neuroscience and Cell Biology, University of Coimbra, Coimbra, Portugal; 3 Department of Medical Sciences and Institute of Biomedicine – iBiMED, University of Aveiro, 3810-193 Aveiro, Portugal

**Keywords:** Immune evasion, Parasite immune evasion, Inflammation

## Abstract

The protozoan *Giardia lamblia* is the most common cause of parasitic gastrointestinal infection worldwide. The parasite developed sophisticated, yet not completely disclosed, mechanisms to escape immune system and growth in the intestine. To further understand the interaction of *G. lamblia* with host immune cells, we investigated the ability of parasites to modulate the canonical activation of mouse macrophages (Raw 264.7 cell line) and human monocyte-derived macrophages triggered by the TLR4 agonist, lipopolysaccharide (LPS). We observed that *G. lamblia* impairs LPS-evoked pro-inflammatory status in these macrophage-like cells through inhibition of cyclooxygenase-2 and inducible nitric oxide synthase expression and subsequent NO production. This effect was in part due to the activity of three *G. lamblia* proteases, a 135 kDa metalloprotease and two cysteine proteases with 75 and 63 kDa, that cleave the p65^RelA^ subunit of the nuclear factor-kappa B (NF-κB). Moreover, *Tnf* and *Ccl4* transcription was increased in the presence of the parasite. Overall, our data indicates that *G. lamblia* modulates macrophages inflammatory response through impairment of the NF-κB, thus silencing a crucial signaling pathway of the host innate immune response.

## Introduction

*Giardia lamblia* (syn. *G. intestinalis*, *G. duodenalis*) is a flagellated protozoan parasite that infects and colonizes small intestine causing diarrhea in a wide variety of mammalian species^1^. It is the most common pathogenic parasitic infection in humans, with an estimated 280 million cases of symptomatic giardiasis annually worldwide^2^. The transmission of the parasite usually occurs after ingestion of infectious cysts via contaminated water or food. Giardiasis is characterized by a wide range of clinical manifestations, from asymptomatic to vomiting, abdominal pain, weight loss, severe diarrhea and malabsorption syndrome. Regardless of these clinical symptoms, giardiasis in human is typically characterized by slight or no mucosal inflammation (Reviewed in^3,4^).

Both branches of the immune response, innate and adaptive immunities, are essential for the clearance of *Giardia*^5^. Over the past five years, multiple works have shown that the eradication and development of protective immunity against *G. lamblia* is dependent not only on B cell-mediated antibody production and T cell-mediated immune responses^6^ but also on the induction of an interleukin 17 A (IL-17A) intestinal response^7–9^. It is now well-known that upregulation of IL-17A is needed for the release of IgA into the lumen of the intestine^9,10^, for the production of antimicrobial peptides, in the regulation of complement activation^9^, being of utmost relevance during acute symptomatic *Giardia* infections in humans^8^.

Surprisingly, epithelial cells, when exposed to parasites *in vitro* produce cytokines that are chemotactic for immune cells being therefore anticipated an increase in inflammatory status^11^. However, *Giardia* parasites subvert and limit the inflammatory response in small intestine allowing its effective colonization^12^. For instance, *Giardia* trophozoites were shown to avoid host immune responses by hindering nitric oxide (NO) production in human intestinal epithelium cells^13^, limiting dendritic cell production of the pro-inflammatory cytokine IL-12^14^, and suppressing macrophages expression of IL-8 and GRO^15^. Despite these studies, data regarding the molecular mechanisms by which *Giardia* parasites modulate innate immune cells of intestinal mucosa such as macrophages remain scarce.

Macrophages are crucial cells of the innate immune system, being equipped with set of highly conserved pattern recognition receptors (PRRs) that sense microorganisms or microorganism components (commonly designated pathogen-associated molecular patterns (PAMPs)). The engagement of the PAMP with the respective PRR results in the production of cytokines, chemokines, prostaglandin E_2_ (PGE_2_) and NO, pro-inflammatory mediators that are essential to orchestrate an effective immune response^16^. The expression of these pro-inflammatory molecules is tightly regulated by a complex network of intracellular signalling pathways and transcription factors. Among these signalling cascades, mitogen-activated protein kinases (MAPKs) and the transcription nuclear factor-κB (NF-κB) assume a decisive role during infection^17^. The activation of NF-κB occurs upon phosphorylation of the protein κB (IκB) by IκB kinase (Iκκ). The activated NF-κB is rapidly translocated into the nucleus triggering the transcription of target genes, such as *NOS2*, *PTGS2*, *TNF*, *IL1B*, and *IL6*. Regarding MAPKs pathways, they comprise three main subfamilies of kinases that are activated by phosphorylation and include the p38 MAPK, c-jun NH2-terminal kinase (JNK) and extracellular signal-regulated kinase (ERK). MAPKs were shown to be closely connected to the regulation of immunity by controlling cell survival and transcription of pro-inflammatory molecules such as CCL5, CXCL9, CXCL10, TNF-α, inducible NO synthase and genes involved in antigen presentation^18,19^.

To survive and evade the protective response of the host cells, several microorganisms manipulate these signaling pathways, changing the balance between positive and negative signals, or by directly inactivating intermediates through cleavage or dephosphorylation^20,21^. This signaling alteration normally evokes an increase in the production of immunosuppressive molecules, such as IL-10 and/or a decreased expression of proinflammatory mediators, such as IL-12, IFN-γ and NO.

Proteolytic cleavage of key inflammatory regulators is an effective strategy to circumvent or neutralize the host immune system thus facilitating the colonization by the microrganisms^20,22^. Consequently, the study of the proteolytic enzymes produced by parasites has attracted considerable interest by the scientific community since they can mediate crucial events of host-pathogen interaction.

It is well documented that *G. lamblia* trophozoites contains multiple proteases^23–27^, some of them demonstrating relevance in giardiasis pathogenesis^28,29^. Recent studies demonstrated that the secretion of cathepsin B cysteine proteases by *G. lamblia* infections attenuate neutrophil/ polymorphonuclear leukocyte (PMN) recruitment^30^. In addition, *G. lamblia* cysteine proteases also induce cleavage and redistribution of the intestinal epithelial cytoskeletal protein villin^31^.

Therefore, in an attempt to disclosed the molecular mechanisms involved in macrophages manipulation by *Giardia lamblia* we investigated the direct interaction of macrophages (Raw 264.7 cell line) and human monocyte-derived macrophages with *G. lamblia* trophozoites, having a special focus on the effects on MAPKs and NF-κB signal transduction pathways. The putative effects of infection on NO production, iNOS and COX-2 expression and cytokine/chemokine transcription were also analyzed. Additionally, the ability of parasites to counteract LPS-evoked macrophage-like cells activation was also disclosed.

## Results

### *Giardia* induces marginal mRNA levels of *Il1b*, *Il6*, *Il10*, *CCL3*, *Ccl4* and *TNF* and slightly affects the LPS-induced transcription of cytokine/chemokine in macrophage-like cells

In response to pathogenic microorganisms, macrophages produce cytokines that will define the nature of T-cell response. The pattern of such immune response is influenced by the balance between the secretion of pro-inflammatory and anti-inflammatory cytokines. Consequently, experiments were performed to examine the effect of *Giardia* trophozoites on RAW 264.7 macrophages cytokine/chemokine transcription and on the ability of parasites to manipulate the LPS-induced cytokine/chemokine profile.

**Figure 1:**
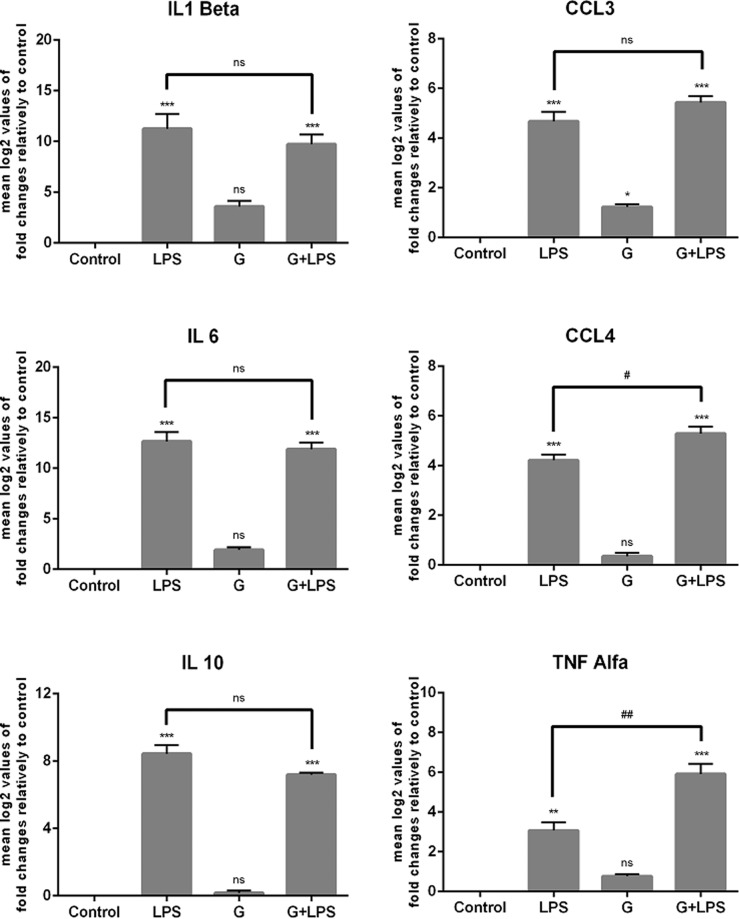
Effect of *Giardia* trophozoites on the expression of cytokines triggered by LPS in murine macrophages. Raw 264.7 cells (1.5 × 10^6^ cells) were maintained in culture medium (control), or pre-incubated with *G. lamblia* (7.5 × 10^6^ cells) for 1 h, and then activated with 1 μg/ml LPS for 6 h. The levels of mRNA were assessed by RT-PCR, for *Il1b*, *Il6*, *Il10*, *Ccl3*, *Ccl4*, and *Tnf-α*. Gene expression is indicated as log2 values of fold changes relatively to control. Each value represents the mean ± SEM. from three independent biological experiments run in duplicate (^*^p < 0.05, ^**^p < 0.01, ^***^p < 0.001, compared to control; ^#^p < 0.05, ^##^p < 0.01 compared to LPS; ns, not significant).

qPCR analyses showed that while LPS treatment results in a significant increase on the transcription of *Il1b*, *Il6*, *Il10*, CCL3,* Ccl44* and *TNF* (p < 0.01; p < 0.001), the interaction with *G. lamblia* had no significant effects on mRNA levels of these molecules, except for chemokine *Ccl3* (p < 0.05) (Fig. 1). In macrophage-like cells cultured with *G. lamblia* and then exposed to LPS we observed a slight decrease in the transcription of *Il1b*, *Il6* and *Il10* and a significant increase in the mRNA levels of *TNF* and *Ccl44* (Fig. 1) (p < 0.001, p < 0.05; respectively).

### *Giardia lamblia* trophozoites strongly impair LPS-induced iNOS expression and nitrite production in macrophage-like cells

**Figure 2:**
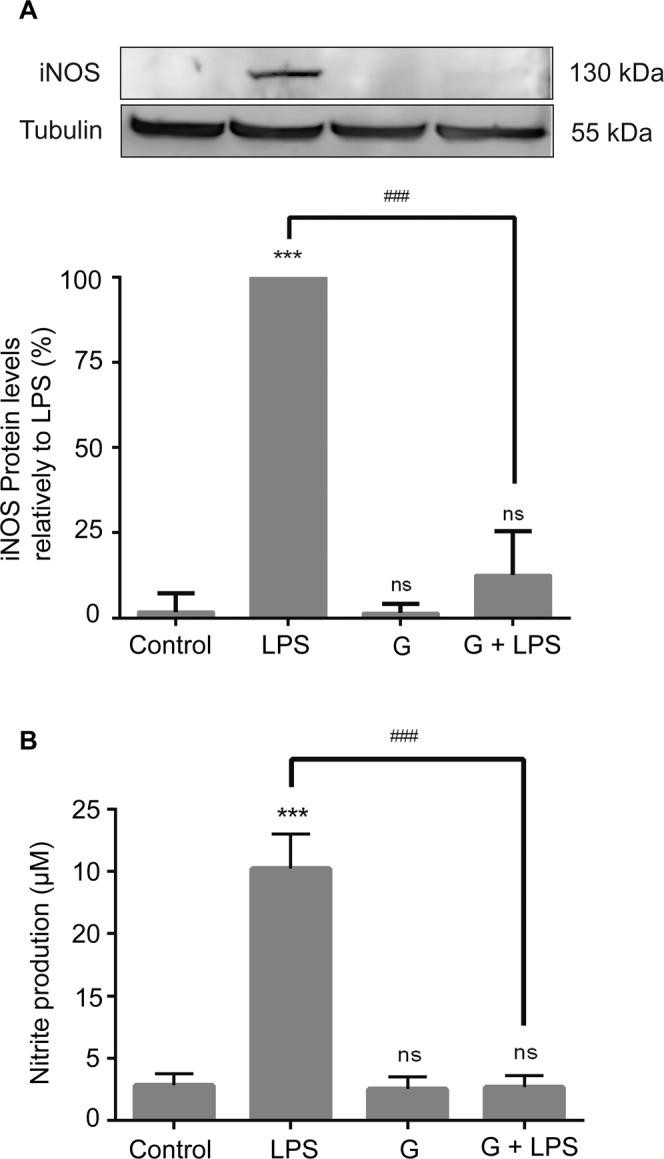
Effects of *Giardia* trophozoites on LPS-induced iNOS protein expression and nitrite production in macrophage-like cells. (**A**) Raw 264.7 cells (6 × 10^5^ cells) were maintained in culture medium (control), or pre-incubated with *G. lamblia* (3 × 10^6^ cells) for 1 h, and then activated with 1 μg/ml LPS for 8 h. iNOS expression was analyzed by Western blot using a specific anti-iNOS antibody and anti-tubulin antibody was used to confirm equal protein loading. The blot shown is representative of 3 blots yielding similar results. Results were expressed as percentage of iNOS protein levels relatively to LPS. (B) Raw 264.7 cells (6 × 10^5^ cells) were maintained in culture medium (control), or pre-incubated with *G. lamblia* (3 × 10^6^ cells) for 1 h, and then treated with 1 μg/ml LPS for 8 h. Each value represents the mean ± SEM from at least 3 independent experiments (^***^p < 0.001, control vs treatment; ^###^p < 0.001, LPS vs treatment; ns, not significant). Full-length blots are presented in Supplementary Fig. S6.

The effect of *G. lamblia* trophozoites on iNOS expression 8 h after cells infection was analyzed by Western blot using a specific anti-iNOS antibody (Fig. 2a). *Giardia* alone was without effect while LPS stimulation of Raw 264.7 cells resulted in a strong increase of the protein iNOS. Surprisingly, LPS-induced expression of iNOS was almost completely abolished by trophozoites (reduction of 87.45 ± 7.45%; p < 0.001).

The effect of trophozoites on LPS-induced NO production in RAW 264.7 cells was also evaluated by measuring nitrite accumulation in macrophage culture medium. In resting conditions, macrophage-like cells produced low levels of nitrites, which increased after LPS stimulation (Fig. 2b). Corroborating the data obtained for iNOS protein expression, interaction of Raw cells with *Giardia* strongly decreased the LPS-induced nitrite production (reduction of 62.2 ± 3.74%; p < 0.001).

### Decrease of LPS-induced NO production is not due to parasite depletion of arginine

*Giardia* consumes arginine as a principal energy source and the reduced availability of this amino acid in medium was shown to lead to decreased production of NO in infected intestinal epithelial cells^13^. In order to verify whether the concentration of arginine could represent a limiting factor to NO production by Raw 264.7 cells we supplemented the medium with 0.4 mM, 10 mM and 20 mM of L-arginine (final concentration 0.8 mM, 10.4 mM and 20.4 mM, respectively).

**Figure 3:**
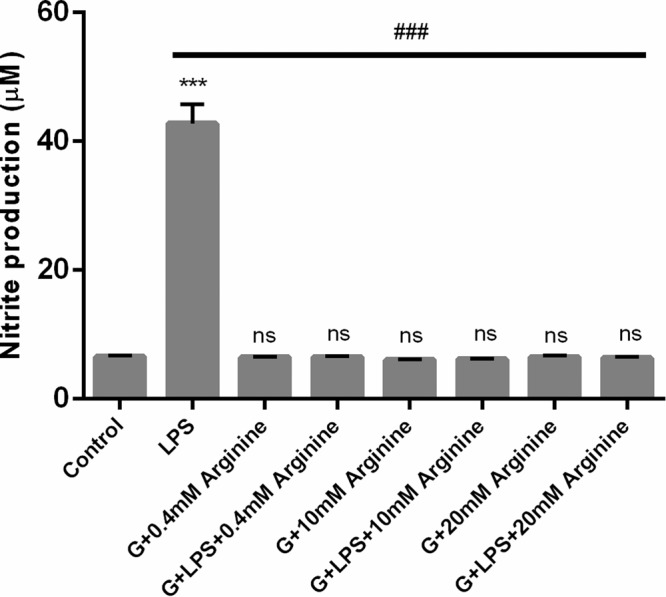
Inhibitory effects of trophozoites on macrophage-like cells nitrite production after supplementation with several arginine concentrations. Raw 264.7 cells (6 × 10^5^ cells) were maintained in culture medium (control), or pre-incubated with *G. lamblia* (3 × 10^6^ cells) for 1 h with DMEM culture medium supplemented with 0.4 mM, 10 mM or 20 mM of L-arginine (final concentration 0.8 mM, 10.4 mM and 20.4 mM), and then activated with 1 μg/ml LPS for 8 h. Each value represents the mean ± SEM from at least 3 independent experiments (^***^p < 0.001, control vs treatment; ^###^p < 0.001, LPS vs treatment; ns, not significant).

The addition of arginine to final concentration of 10 mM is sufficient to restore the capability of macrophage-like cells to produce NO when exposed to *G. lamblia* extracts (Supplementary Fig. S1). In contrast, we observed that the addition of arginine does not modify the decrease of NO production caused by live trophozoites in LPS-treated macrophage-like cells indicating that it not results from depletion of arginine by *Giardia* (Fig. 3).

### *Giardia* counteracts the increase of COX-2 expression elicited by LPS

**Figure 4:**
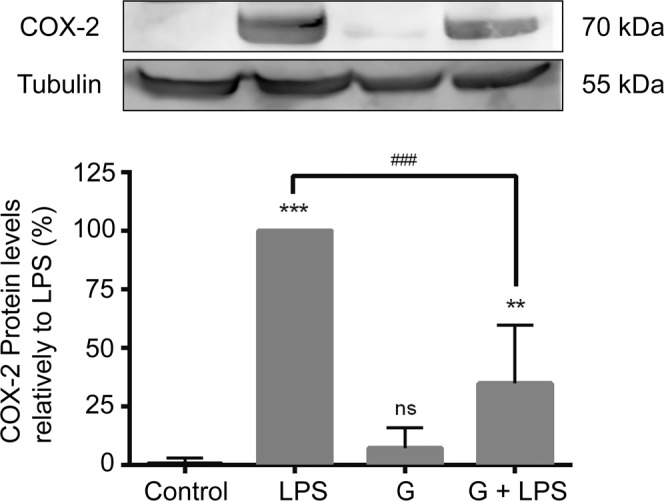
Effects of *G. lamblia* trophozoites on LPS-induced COX-2 protein expression in macrophage-like cells. Raw 264.7 cells (6 × 10^5^ cells) were maintained in culture medium (control), or pre-incubated with *G. lamblia* (3 × 10^6^ cells) for 1 h, and then activated with 1 μg/ml LPS for 8 h. COX-2 expression was analyzed by Western blot using a specific anti-COX-2 antibody and anti-tubulin antibody was used to confirm equal protein loading. The blot shown is representative of 3 blots yielding similar results. Results were expressed as percentage of COX-2 protein levels relatively to LPS. Each value represents the mean ± SEM from at least 3 independent experiments (^**^p < 0.01, ^***^p < 0.001 compared to control; ^###^p < 0.001, LPS vs treatment; ns, not significant). Full-length blots are presented in Supplementary Fig. S7.

We also investigated the effect of *G. lamblia* trophozoites on macrophage-like cell COX-2 expression (Fig. 4). Similarly, to the observed for iNOS expression, LPS treatment caused a strong increase in COX-2 protein levels, being this increase significantly inhibited by *Giardia* parasites (reduction of 65.05 ± 12.38%; p < 0.001). In order to address if this effect also occurred in other cells, we performed the same experiments with primary human monocyte-derived macrophages. As shown in Supplementary Fig. S2, the effect of *G. lamblia* trophozoites over LPS-induced COX-2 expression is maintained in human macrophages.

### *Giardia* does not modify the pattern of p38 MAPK and JNK 1/2 activation but significantly decreases the total levels of NF-κB p65^RelA^ subunit

Next, in order to undercover possible molecular mechanism explaining the observed effects of *Giardia* parasites on macrophages we examined the impact of infection on the activation of MAP kinases and of the transcription factor NF-κB.

**Figure 5:**
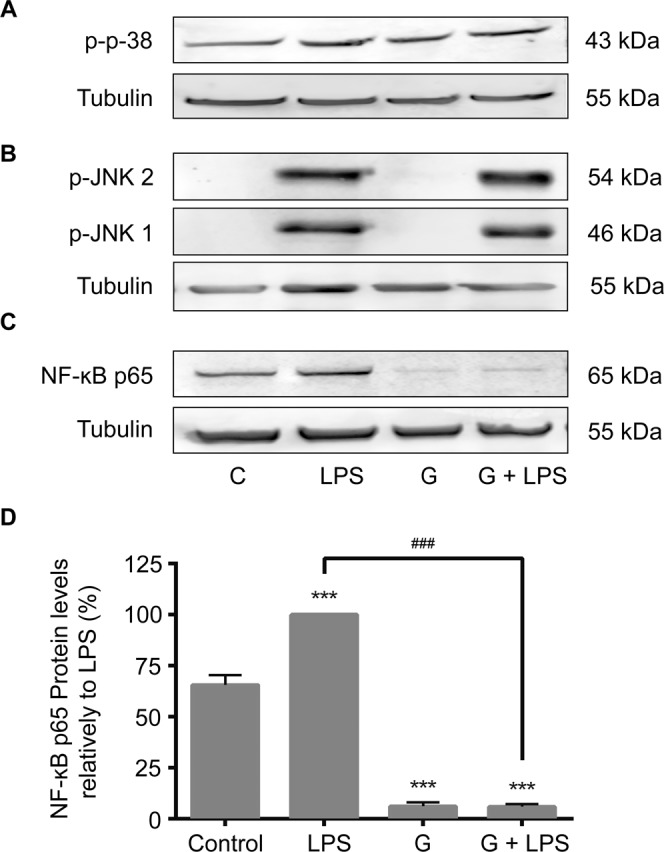
Effects of trophozoites on NF-κB and MAPKs signaling pathways. Raw 264.7 cells (6 × 10^5^ cells) were maintained in culture medium (control), or pre-incubated with *G. lamblia* (3 × 10^6^ cells) for 1 h, and then activated with 1 μg/ml LPS for 30 minutes. Total cell extracts were analyzed by Western blot using antibodies against (**A**) phospho-p38 MAPK, (**B**) phospho-JNK 1/2 and (**C**) NF-κB p65. An anti-tubulin antibody was used to confirm equal protein loading. The blot shown is representative of 3 blots yielding similar results. (**D**) Results were expressed as percentage of NF-κB p65 protein levels relatively to LPS. Each value represents the mean ± SEM from at least 3 independent experiments (^***^p < 0.001, compared to control; ^###^p < 0.001, compared to LPS; ns, not significant). Full-length blots are presented in Supplementary Fig. S8.

**Figure 6:**
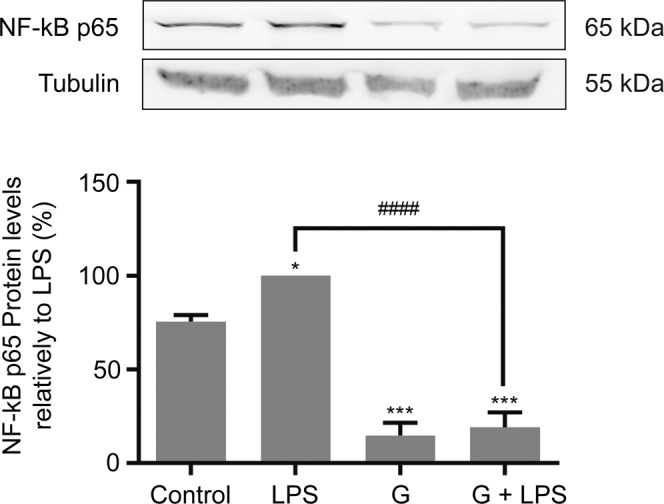
Effects of *Giardia* trophozoites infection of human macrophages on the levels of NF-κB p65 subunit. Cells (6 × 10^5^) were maintained in culture medium (control), or pre-incubated with *G. lamblia* (3 × 10^6^ cells) for 1 h, and then activated with 1 μg/ml LPS for 30 minutes. Total cell extracts were analyzed by Western blot using antibodies against NF-κB p65. An anti-tubulin antibody was used to confirm equal protein loading. Results were expressed as percentage of NF-κB p65 protein levels relatively to LPS. Each value represents the mean ± SEM from at least 3 independent experiments (^***^p < 0.001, compared to control; ^####^p < 0.0001, compared to LPS).

**Figure 7:**
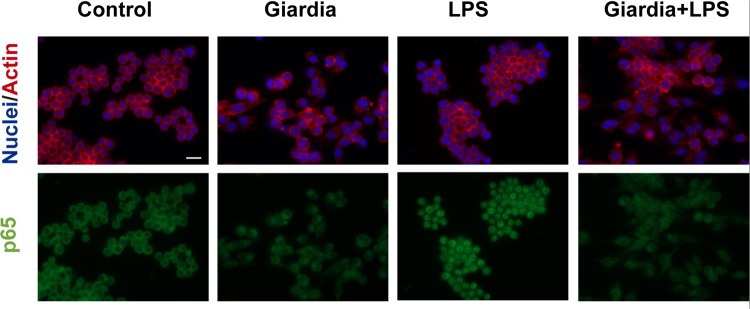
Immunofluorescence analysis of the effect of *Giardia lamblia* trophozoites on NF-κB p65 protein levels and localization. Raw 264.7 cells (5 × 10^4^ cells) were incubated on µ-slides 8 wells and treated as detailed in methods section. The images were acquired with an Axio Observer.Z1 inverted fluorescence microscope (Zeiss). The images shown are representative of three individual experiments yielding similar results. Scale bar = 20 µm.

We found that the parasites *per se* were without effect on the tested MAPKs signaling pathways and do not significantly modify the LPS-induced activation of p38 MAPK and JNK 1/2 (Fig. 5a,b). Surprisingly, the total levels of NF-κB p65^RelA^ subunit were strongly decreased in cells exposed to *Giardia*, independently of the presence of LPS (Fig. 5c). In mouse macrophages stimulated with LPS and infected with trophozoites we observed a strong decrease on the NF-κB p65^RelA^ subunit levels to 0.58 ± 1.10% (p < 0.001). Moreover, *Giardia* alone also decreased p65 protein levels (0.33 ± 1.16%) (Fig. 5d). The level of NF-κB p65^RelA^ subunit were also decreased in human monocyte-derived macrophages exposed to *Giardia* either in the absence or in the presence of LPS (Fig. 6), confirming the results previously obtained in mouse macrophages. These results were confirmed by immunofluorescence microscopy experiments showing that co-incubation of *G. lamblia* trophozoites with macrophage-like cells resulted in a down-regulation of NF-κB p65^RelA^ subunit and a redistribution of cytoskeletal proteins (Fig. 7). It is noteworthy that macrophage-like cells viability was not affected by *G. lamblia* trophozoites or extracts (30 min and 8 h after cells infection), in the presence or in the absence of LPS, compared against control (Supplementary Fig. S3).

### Decrease of NF-κB p65^RelA^ subunit levels is contact dependent

In order to determine whether the effects of *G. lamblia* trophozoites on NF-κB p65^RelA^ subunit, iNOS and COX-2 occur via direct contact with macrophage-like cells or via excretory-secretory products (ESP), we analyzed the interaction of trophozoites with cells using a transwell co-culture system.

**Figure 8:**
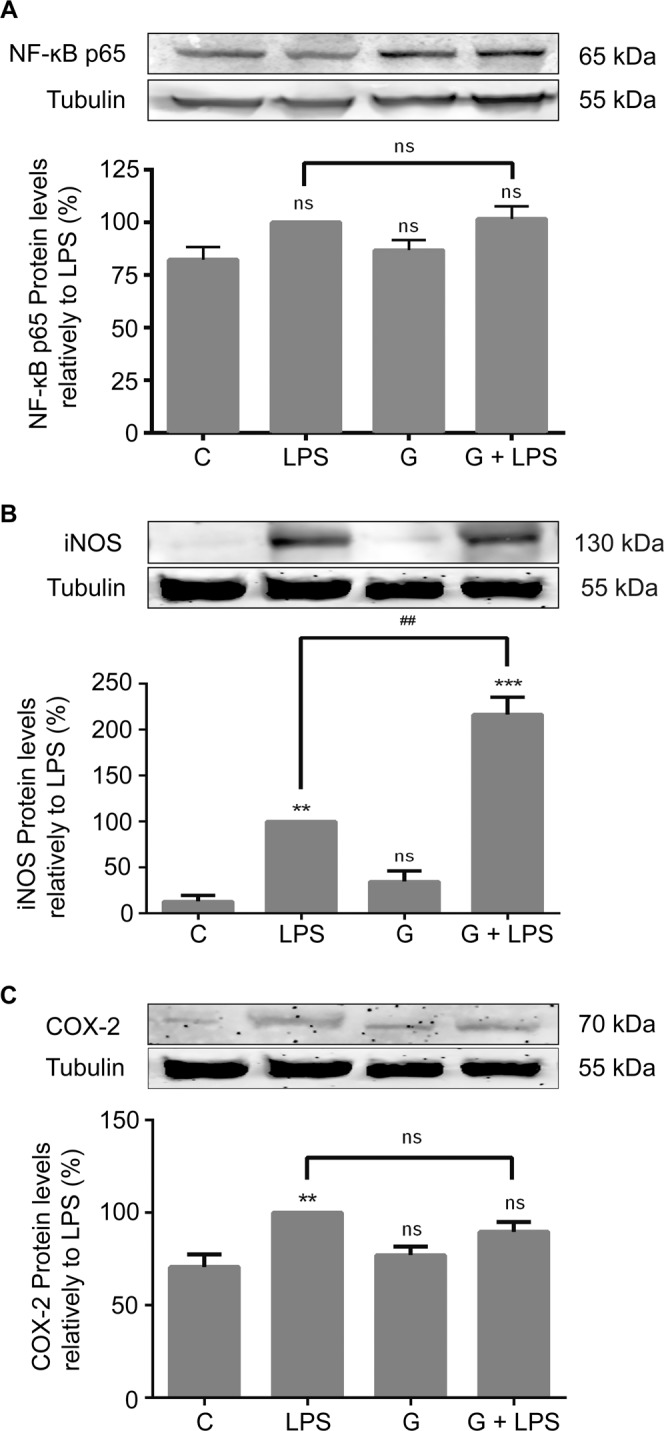
Effects of trophozoites excretory-secretory products on the total levels of NF-κB p65, iNOS and COX-2. Trophozoites of *G. lamblia* were grown on the upper surface of a 6-well inserts and macrophage-like cells were seeded on the bottom of the wells. Raw 264.7 cells (3 × 10^6^ cells) were maintained in the culture medium (control), or pre-incubated with *G. lamblia* trophozoites (1.5 × 10^6^ cells) for 1 hour, and then activated with 1 μg/ml LPS for 30 min (NF-κB p65) or 8 h (iNOS and COX-2). Total cell extracts were analyzed by Western blot using antibodies against (**A**) NF-κB p65^RelA^, (**B**) iNOS and (**C**) COX-2. An anti-tubulin antibody was used to confirm equal protein loading. The blot shown is representative of 3 blots yielding similar results. Each value represents the mean ± SEM from at least 3 independent experiments (^**^p < 0.01, ^***^p < 0.001, compared to control; ^##^p < 0.01, compared to LPS; ns, not significant). Full-length blots are presented in Supplementary Fig. S9.

*G. lamblia* trophozoites were grown on the upper surface of 6-well inserts and Raw cells were seeded on the bottom of the wells. Macrophage-like cells were treated with LPS and NF-κB p65^RelA^, iNOS and COX-2 protein levels were analyzed by Western blot. No alterations were detected on the total levels of the three proteins (Fig. 8), thus suggesting that p65, iNOS and COX-2 disappearance is dependent on the direct contact between macrophage-like cells and parasites. Furthermore, the effects of *G. lamblia* extract on macrophage-like cells corroborate with this conclusion (Supplementary Fig. S4).

### NF-κB p65^RelA^ subunit is cleaved by *Giardia lamblia* cysteine and metalloproteases

**Figure 9:**
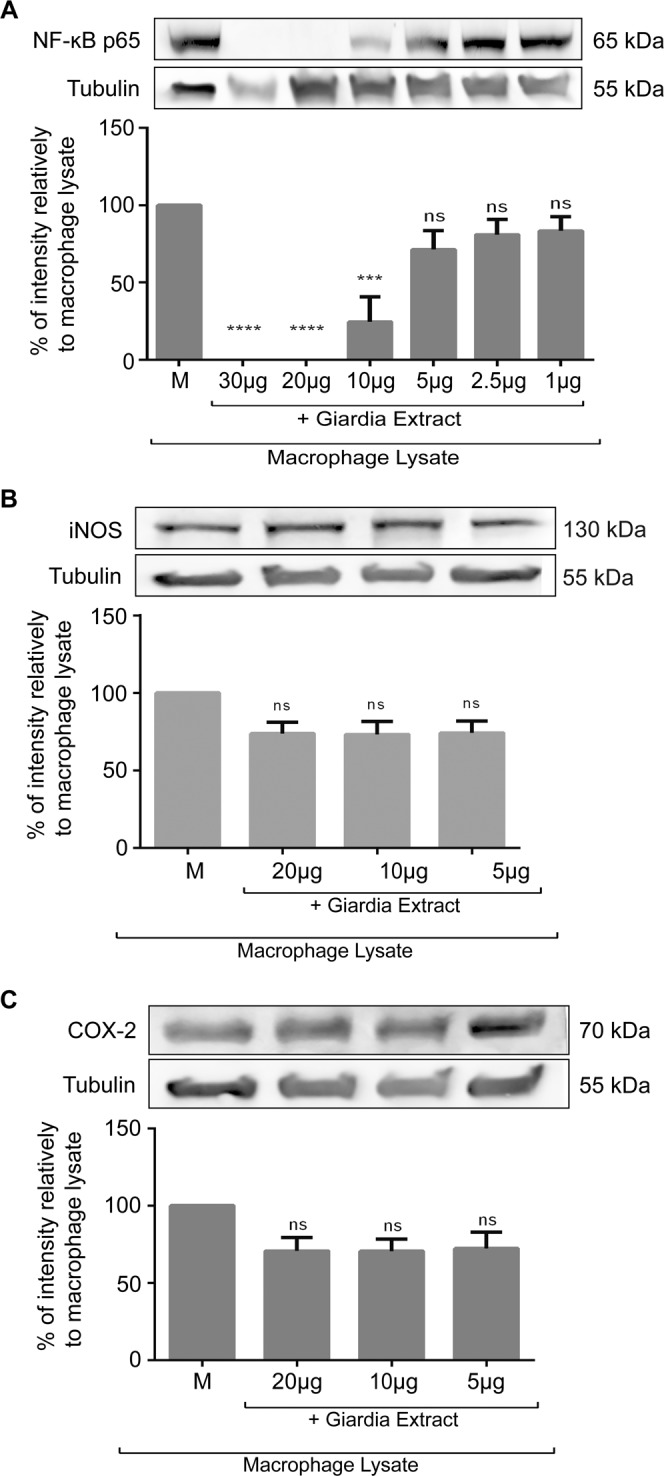
Proteolytic activity of *Giardia lamblia* extracts over NF-κB p65^RelA^, iNOS and COX-2. LPS-activated macrophage-like cells lysates were incubated with *G. lamblia* extracts in several concentrations (30 µg, 20 µg, 10 µg, 5 µg, 2.5 µg and 1.0 µg). NF-κB p65^RelA^ (**A**), iNOS (**B**) and COX-2 (**C**) levels were analysed by Western blot using specific anti-antibodies and anti-tubulin antibody. The blot shown is representative of three blots yielding similar results. Each value represents the mean ± SEM from at least 3 experiments (^***^p < 0.001, ^****^p < 0.0001, compared to LPS-activate macrophage-like cells; ns, not significant). Full-length blots are presented in Supplementary Fig. S10.

We further addressed the direct effects of *Giardia* protein extracts on the levels of LPS-evoked inflammatory proteins in particular of iNOS, COX-2 and NF-κB p65^RelA^. For this, lysates of LPS-activated macrophage-like cells (40 µg) were treated for 1 h with different amounts of parasite extracts (30 µg, 20 µg, 10 µg, 5 µg, 2.5 µg and 1 µg) (Fig. 9).

As expected, LPS stimulation of macrophage-like cells resulted in a strong NF-κB p65^RelA^ subunit protein expression (Fig. 9a). Strikingly, levels of NF-κB p65^RelA^ were strongly decreased in the presence of *Giardia* extracts being the effects significant for 10 µg (p < 0.001), 20 µg (p < 0.0001) and 30 µg (p < 0.0001).

Similarly to the observed for NF-κB p65^RelA^, treatment of Raw 264.7 cells with LPS caused a strong increase in the expression of iNOS (Fig. 9b) and COX-2 (Fig. 9c) in. However, contrarily to the observed for NF-κB p65^RelA^, exposure of cell lysates to *Giardia* extracts does not have significant effects on the levels of these proteins.

To determine whether the effects of *Giardia* on NF-κB p65^RelA^ subunit occurs via degradation by parasite proteases, the proteolytic activity of *G. lamblia* was analysed by Zymography using gelatin (0.1%) or BSA (0.1%) as substrates. Proteinase activity was only observed on gelatin gels in concentrations above 10 µg (Supplementary Fig. S5). The zymogram revealed proteolytic activity, distributed in the migration region of 135 to 63 kDa. Among the profiles, the main pronounced zones of proteolysis were distinguished at 100, 75 and 63 kDa.

The characterization of the *Giardia* proteases evolved on gelatinolytic activity was based on inhibition assays employing inhibitors for the main classes of proteases (cysteine, serine, metallo and aspartic proteases). The effect of inhibitors was evaluated by gelatin zymography using 10 µg of *Giardia* extracts. Samples were incubated with protease inhibitors: EDTA, PHEN, E-64, LEUP, PEPA, PEPAP, PMSF and AEBSF.

**Figure 10:**
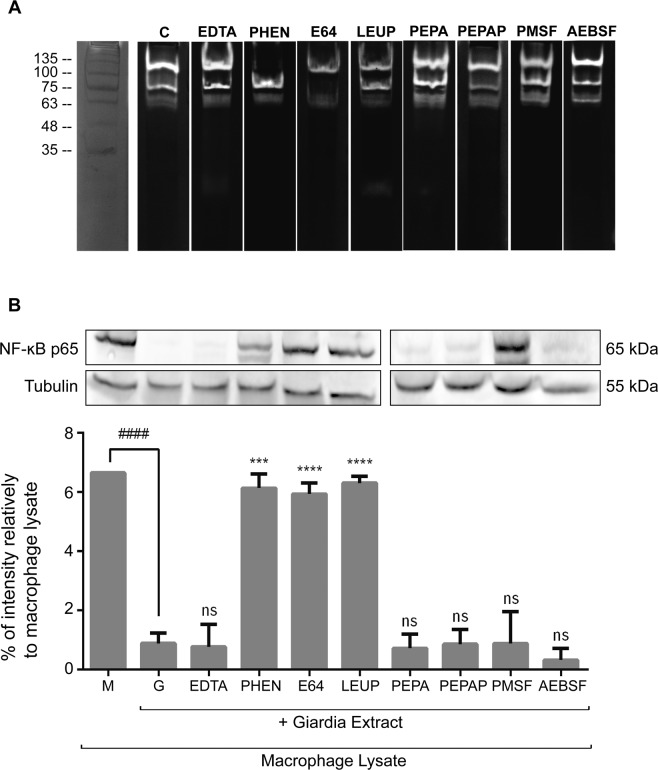
Effects of protease inhibitors on *Giardia lamblia* protease activities and *Giardia*-promoted NF-κB p65 protein degradation. (**A**) Zymograms of *Giardia* lysate 10 µg (C; control of proteolytic activity) with protease inhibitors EDTA (5 mM), PHEN (10 mM), E-64 (10 μM), LEUP (50 µM), PEPA (1 μM), PEPAP (1 µM), PMSF (1 mM), and AEBSF (1 mM). Molecular mass markers are shown on the left in kilodaltons (kDa). (**B**) LPS-activated macrophage-like cells lysates were incubated with *G. lamblia* extracts (10 µg) and with protease inhibitors EDTA, PHEN, E-64 and LEUP, PEPA, PEPAP, PMSF and AEBSF. NF-κB p65 subunit levels were analyzed by Western blot using a specific anti-p65 antibody and anti-tubulin antibody. The blot shown is representative of three blots yielding similar results. Each value represents the mean ± SEM from at least 3 experiments (^****^p < 0.0001, LPS-activated macrophage-like cells *versus Giardia* treated extracts; ^###^p < 0.001, ^####^p < 0.0001, inhibitors *versus Giardia*; ns, not significant). Full-length blots are presented in Supplementary Fig. S11.

As observed previously, the *Giardia* proteolytic activity was visible at a range from 135 to 63 kDa (Fig. 10a) with prominent bands observed at 100, 75 and 63 kDa. The proteolytic activities were mainly blocked by E-64 (cysteine protease inhibitor) at 100, 75 and 63 kDa; LEUP (cysteine-serine protease inhibitor) at 100 and 75 kDa; and PHEN (metalloprotease inhibitor) at 100 kDa.

To determine whether the effects of *Giardia* on NF-κB p65^RelA^ subunit occur via degradation by parasite proteases, we addressed the interaction of *Giardia*- macrophage-like cells in the presence of protease inhibitors. The effect of protease inhibitors on NF-κB p65^RelA^ proteolysis was studied using *Giardia* extracts (10 µg) and LPS-activated macrophage-like cells lysates (30 µg), pre-incubated with protease inhibitors: EDTA, PHEN, E-64, LEUP, PEPA, PEPAP, PMSF and AEBSF (Fig. 10b). As expected, the interaction of LPS-activated cell lysates with *Giardia* extract results on a significant decrease of NF-κB p65^RelA^ (p < 0.0001). When protease inhibitors were used, NF-κB p65^RelA^ proteolysis was significantly reversed by the metalloprotease inhibitor PHEN (p < 0.001), the cysteine inhibitor E-64 (p < 0.0001) and the cysteine-serine inhibitor LEUP (p < 0.0001).

## Discussion

In most *Giardia* infections, clinical manifestations are self-limiting, with transient intestinal complications. However, infections may also last for several months/years as chronic^5,32,33^ or as asymptomatic infections (the majority in endemic areas). In these cases, the parasite is likely to subvert host’s immune system.

The precise nature of parasite-immune cells interactions during giardiasis has significant consequences for both immunopathology and immunity. Few researches have been focused in this area and consequently the molecular basis characterizing the modulation of innate immune system by *Giardia* parasites remain to be deeply explored^15,30,31^. These facts prompted us to study the interaction between *G. lamblia* and macrophage-like cells (Raw 264.7 cell line) at multiple molecular levels. Our data demonstrated that *G. lamblia* does not trigger in macrophage-like cells the activation of canonical pro-inflammatory signaling cascades such as MAPKs and NF-κB, being this reflected by minimal induction of iNOS, COX-2 and of the transcription of cytokines/chemokines such as TNF-α, CCL3 and CCL4. These results correlate well with data showing limited intestinal inflammation during giardiasis. Indeed, besides the highly variable clinical manifestation of giardiasis between individuals, it is well-known that infection with *G. lamblia* does not cause overt inflammation of the intestinal mucosa and most of the infected individuals had asymptomatic giardiasis (Reviewed in^3,4^).

Previous works have shown that *Giardia* can actively down-regulate inflammatory signals in epithelial and immune cells in order to be able to survive and colonize small intestine. The parasite was shown to rescue NO production in human epithelial cells by competing with NO synthase for its substrate arginine^13,34^. Additionally, it was proposed that inhibition of NO production by *G. lamblia* may also result from the expression of flavohemoglobin protein (flavoHb)^35^. This protein has a well-known NO reductase activity converting NO to nitrate, protecting that way the parasite from nitrosative stress^35^. Regarding direct interaction with immune cells, Kamda and Singer^14^ showed that *Giardia* parasites inhibit dendritic cells production of the pro-inflammatory IL-12 by a mechanism dependent on the negative regulation exerted by the PI3K/Akt pathway over Toll-like receptors (TLR) signals. Furthermore, it was observed that *Giardia* parasites could evoke the suppression of the host immune response by stopping the secretion of the pro-inflammatory cytokines IL-8 and GRO, and increasing the caspase-3 activity in Caco-2 cells, reducing in that way the cell number through apoptosis^15^.

In order to explore possible mechanism of *Giardia* to counteract inflammatory signals in macrophages we performed a series of experiments where Raw 264.7 cells were co-incubated with parasite along with the TLR4 ligand LPS. Surprisingly, we observed that parasites actively counteract the LPS-induced expression of COX-2, iNOS and downstream NO production. It is well documented that *G. lamblia* consumes arginine as an energy source^13,36^ and secretes enzymes of the arginine metabolic pathway (arginine deiminase (ADI) and ornithine carbamoyl transferase (OCT)), upon interaction with intestinal epithelial cells^37^. These two enzymes are able to metabolize arginine to produce ATP thus decreasing the amount of arginine. To disclose whether the decrease of NO production detected in macrophage-like cells infected with *Giardia* was due to a reduction in the amount of available arginine, we supplemented the culture medium with the amino acid. It has already been reported that addition of external arginine (concentration of 0.4 mM) can partially restore the NO production of parasite-interacted human intestinal epithelial cells^34^. Under these experimental conditions the decrease caused by parasites persisted indicating that suppression of iNOS expression rather than depletion of arginine is the main mechanism.

The downregulation and/or up-regulation of iNOS expression by *Giardia* trophozoites infection has been observed *in vivo* and *in vitro* assays. Accordingly, Stadelmann and colleagues^34^ investigated the interaction of *Giardia* trophozoites with human differentiated Caco-2 cells and observed that parasite has both iNOS inducing and suppressive actions. Similar results also detected by Mokrzycka *et al*.^38^ studying *Giardia* human infections. The expression of iNOS in duodenal biopsies of children with giardiasis was as high as in control group, though in others it was not noticeable. Maloney *et al*.^39^ observed an increase of ARG1 and iNOS expression in mouse intestine following infection.

Probably, regulation of macrophage-released COX-2-dependent PGE_2_ levels is a strategy through which *Giardia* promotes favorable conditions that enable their permanence in the host. Modulation of COX-2 expression has also been observed in *Leishmania*^40^ and *Trypanosoma cruzi* parasites^41,42^ as an immune escaping mechanism. However, unlike *G. lamblia* trophozoites, *Leishmania* parasites induce COX-2 expression, which signals to an increased PGE_2_ production, with subsequent down-regulation of macrophage IL-12 production (Reviewed in^43^).

Regarding the effects of *Giardia* over LPS-induced cytokine/chemokine production we observed that while *Il1b*, *Il10*, *Il6* and *Ccl3* transcription was unaffected, *T*nf and *Ccl4* mRNA levels presented a small increase (p < 0.01 and p < 0.05, respectively). These results are consistent with previous studies showing an infection-dependent bordering increase in TNF-α secretion while IL-6 was slightly decreased^14,44,45^. Accordingly, Lee and colleagues^44^ verified that *Giardia* trophozoites induce TNF-α production in human gastrointestinal cells whereas IL-6 release was scarcely detected. Similar results were also observed in other cell types. Indeed, *G. lamblia* induce small amounts of IL-6 and TNF-α in murine bone marrow-derived dendritic cells^14^ and in mast cells, *Giardia* soluble antigens up-regulate the secretion of IL-6 and TNF-α, however only a moderate rise on these two cytokines was observed after infection with live trophozoite^46^. Therefore, the low status of mast cells activation in the presence of live trophozoite may be the result of an efficient evasion strategy triggered by parasites.

The expression and production of inflammatory mediators by immune cells is under tight control of a complex network of intracellular signaling pathways and transcription factors. As part of their pathogenic strategies, several microorganisms manipulate these signaling cascades in host cells in order to inflammation and to evade the activities of effector immune cells^20,21^. Therefore we analyzed the effects of *Giardia* parasites on the levels of LPS-triggered activation of p38 MAPK, JNK 1/2 and NF-κB subunit p65, three central players in inflammatory signaling pathways. While parasites were without effect on p38 and JNK1/2, they strikingly decrease the total levels of the p65^RelA^ NF-κB subunit, either in presence or in absence of LPS.

As a recent study showed that *G. lamblia* cathepsin cysteine proteases induce villin breakdown in a contact-dependent manner^31^, we performed additional experiments to address if the observed decreases in the iNOS, COX-2 and p65^RelA^ protein levels were due to a secreted parasitic component or dependent on direct contact. In our experimental model, the decrease of total levels of the three proteins occurred via direct contact with macrophage-like cells and was not mediated by parasite excretory-secretory products. Therefore, we could hypothesize that *G. lamblia* trophozoites induces the cleavage of the p65^RelA^ NF-κB subunit resulting in the observed attenuation of LPS-induced COX-2 and iNOS expression. In accordance, the inactivation/cleavage of NF-κB signaling effectors has been shown to be an immune evasion approach shared by multiple microorganisms. For instance, *Leishmania mexicana* amastigotes induce a proteolytic degradation of NF-κB, ERK and JNK in infected macrophages through a cysteine peptidase B^47^. Moreover, *Leishmania* promastigotes were shown to infect macrophages and dendritic cells and to cleave the p65^RelA^ subunit resulting in a p35^RelA^ fragment able to evoke transcriptional activity^48,49^. The cleavage was attributed to the *Leishmania* surface metalloprotease GP63, a virulence factor previously implicated in the activation of several host protein such as TCPTP, SHP-1 and PTP-1B which in turn dephosphorylate IRAK-1, JAK/STAT, and MAPKs^50^. The authors postulated that the resultant NF-κB cleavage fragment p35^RelA^ may represent a crucial mediator through which *Leishmania* promastigotes induce several chemokines without inducing other NF-κB-regulated genes that are unfavorable for parasite survival such as *Ptgs2* (coding for COX-2) and *Nos2* (coding for iNOS). Our results support a similar phenomenon, where p65^RelA^ inactivation/modification by *Giardia* causes the impairment of *Nos2* and *Ptgs2* transcription without compromising the levels of chemokines such as CCL3. Mechanisms aiming the manipulation of NF-κB activity are also common in other pathogens. For instance, the bacteria *Yersinia* spp. was shown to produce a ubiquitin hydrolase that is injected into the macrophage cytoplasm and reverses ubiquitination of IκB which results in NF-κB inhibition^51^. A recent report documents a type III effector protease NleC from enteropathogenic *Escherichia coli* that signals NF-κB for degradation^52^. Additionally, Christian and colleagues^53^ demonstrated that cleavage of the p65 by the chlamydial protease-like activity factor spoils proinflammatory signaling in *Chlamydia*-infected cells. All this data indicates that NF-κB signaling cascade, being a central player in innate immunity and inflammation, is also a preferential target for pathogens in order to circumvent deleterious immune responses.

The results herein gathered led us to hypothesize that *Giardia* may contain proteases that directly cleave NF-kB p65^RelA^ causing its inactivation. Since it was already demonstrated that *G. lamblia* trophozoites degrade CXCL8 through a secreted cathepsin B protease^30^, we decided to check this hypothesis. Our results showed that *G. lamblia* extracts possess high proteolytic activity over p65^RelA^, but not on iNOS or COX-2. Zymograms revealed that *Giardia* trophozoites contain at least three distinct proteases, a 135 kDa metalloprotease and two cysteine proteases with 75 and 63 kDa. Moreover, the cleavage of NF-κB p65^RelA^ was blocked by phenantroline, E-64 and leupeptin, selective inhibitors of metalloprotease and cysteine proteases, respectively. Likewise, several studies reported the blockade of *Giardia* gelatin hydrolysis by cysteine and serine proteases inhibitors as E-64 and leupeptin^25,54,55^. As in *Giardia* trophozoites cysteine proteases are highly abundant, it was reasonable that the main proteolytic activity observed in our experiments was due to proteases of this class.

There are several mechanisms through which the microorganism proteases could enter into the cytosol in order to cleave NF-kB. Recent studies suggest that extracellular vesicles (EVs) are used by parasites to deliver contents into the host cells, constituting a mechanism by which they manipulate immune response. Numerous evidence indicate that proteins secreted by parasites are enclosed within exosomes and recent studies on *Schistosoma japonicum*, *Schistosoma mansoni, Trypanosoma cruzi*, *Toxoplasma gondii* and *Leishmania* suggest that parasites may use exosomes to release proteins into host cells, which could indeed be considered as potential virulence factors^56^. Exosomes could accelerate parasitic infection by inhibiting pro-inflammatory cytokines. Accordingly, *Leishmania* produce exosomes carrying metalloprotease GP63 that has been shown to be essential for establishing and sustaining the infection, concomitantly triggering the cleavage of NF-κB p65^RelA^ subunit in *L. mexicana* and *L. infantum*-infected dendritic cells and macrophages^57^. There is also evidence that *Giardia* is capable of shedding EVs which are involved in pathogen interactions via immunomodulation and trophozoite persistence^58^. Preliminary data generated in our lab point that *Giardia* EVs decrease NF-kB p65 levels in macrophages-like cells (*data not shown*) suggesting that these *Giardia* EVs could contain pathogen effectors such as the proteases identified in the present work. The endocytosis of these vesicles by phagocytic cells may then promote important events such as the impairment of intracellular signalling pathways, like NF-kB pathway.

Overall, this study demonstrated for the first time, that *Giardia lamblia* can differentially target NF-κB in order to modulate the inflammatory functions of macrophage cells. Our results evidence that the impairment of NF-κB transcription factor is a crucial strategy through which parasites limit COX-2 and iNOS expression and downstream NO production. The knowledge of the intracellular signaling profile modulated by *Giardia* parasites in host immune cells highlight putative molecular targets for further development of new therapeutic strategies against giardiasis.

## Methods

### Materials

Dulbecco’s modified Eagles’s medium (DMEM), dimethyl sulfoxide (DMSO), L- arginine, leupeptin (LEUP), L-trans-epoxysuccinyl-leucylamido-(4-guanidino)-butane (E-64), pepstatin A (PEPA), pepA-penetratin (PEPAP), lipopolysaccharide (LPS) from *Escherichia coli* (serotype 026:B6), penicillin and streptomycin were obtained from Sigma Chemical Co. (St. Louis, MO, USA). Fetal bovine serum (FBS) was purchased from Invitrogen (Paisley, UK). The protease and phosphatase inhibitor cocktails were obtained from Roche (Mannheim, Germany). Phenylmethylsulfonyl fluoride (PMSF), ethylenediamine tetraacetic acid (EDTA), 1,10-phenanthroline (PHEN) and 4-(2-aminoethyl)-benzenesulfonyl fluoride (AEBSF) were purchased from Calbiochem (Darmstadt, Germany). Bicinchoninic acid protein assay (BCA) was from ThermoFisher Scientific (Rockford, IL, USA). Antibodies against phospho-p38 MAPK, phospho-JNK, and NF-κB p65^RelA^ were from Cell Signaling Technologies (Danvers, MA, USA). The iNOS antibody was from R&D Systems (Mineapolis, MN, USA) and COX-2 was from Abcam (Cambridge, UK). The anti-tubulin antibody was purchased from Sigma Chemical Co. (St. Louis, MO, USA). The alkaline phosphatase-linked secondary antibodies and the enhanced chemifluorescence (ECF) reagent were obtained from GE Healthcare (Chalfont St. Giles, UK), and the polyvinylidene difluoride (PVDF) membranes were from Millipore Corporation (Bedford, MA). RNAzol reagent, iScript Select cDNA Synthesis kit and Sso Fast Eva Green Supermix were purchased from BioRad (Hercules, CA, USA). Primers were obtained from MWG Biotech (Ebersberg, Germany). All other reagents were from Sigma Chemical Co. (St. Louis, MO, USA) or from Merck (Darmstadt, Germany). The  µ-slides 8 wells and fluorescent mounting medium were purchased from IBIDI GmbH, Germany.

### Raw 264.7 Cell culture

The mouse macrophage cell line Raw 264.7 (ATCC number: TIB-71) was cultured in DMEM supplemented with 10%FBS, 100 U/mL penicillin, and 100 μg/mL streptomycin at 37 °C in a humidified atmosphere of 95% air and 5% CO_2_, as previously described^59^.

### Human monocyte-derived macrophages culture

To obtain human monocytes, peripheral blood mononuclear cells (PBMCs) were firstly isolated by Ficoll-Paque density gradient centrifugation from buffy coats of healthy volunteers provided by the Portuguese Blood Institute. Monocytes were isolated by positive selection using CD14 antibody-coated magnetic microbeads (Miltenyi), as described by the manufacturer. Monocytes (1 × 10^6^ cells/mL) were then cultured in 6-well microplates in RPMI medium supplemented with 10% heat-inactivated fetal bovine serum and 100 ng/mL GM-CSF, which induce their differentiation into macrophages. The medium was changed every two days and macrophages were collected at 7^th^ day culture.

### *Giardia lamblia* trophozoites culture

The culture of *G. lamblia* trophozoites (strain WB, clone 6 (ATCC 30957)) was maintained as previously described^60^. Trophozoite forms were growth in axenic culture at 37 °C in 10 ml of Keister’s modified TYI-S-33 medium. Penicillin (100U/ml) and streptomycin (100 μg/ml) were added during routine culture. Trophozoites of cultures in logarithmic growth phase were used as inoculum to study *G. lamblia* interaction with the macrophage-like cells. Parasites were collected by cooling of the culture vials on ice for 20 min and centrifuged at 400 *g* for 5 min at 4 °C.

### Evaluation of cell viability by MTT assay

To quantify the viable macrophage cells, 3-(4,5-dimethylthiazol-2-yl)−2,5-diphenyl tetrazolium bromide (MTT) reduction colorimetric assay was performed^61^. After macrophage-like cells-*Giardia* interaction, a MTT solution (5 mg/ml in PBS) was added to the wells and cells incubated at 37 °C for 15 min, in a humidified atmosphere of 95% air and 5% CO_2,_ as previously described^59^. Supernatants were then removed and dark blue crystals of formazan solubilized with DMSO. Quantification of formazan was performed using an ELISA automatic microplate reader (Synergy™ HT, BioTek) at 530 nm. The experiments were conducted in triplicate and cell viability was expressed as percentage of control.

### *Giardia lamblia* trophozoite extracts

To obtain the *Giardia* extracts, trophozoites in log-phase of growth were pooled by centrifugation at 400 *g* for 5 min at 4 °C, as previously described^54^. The pellet was then suspended in 0.25 M sucrose and washed twice by centrifugation. Cell suspension were stored at −80 °C during 1–2 days, thawed at room temperature and disrupted at 4 °C by ten periods of 10 s of ultrasonic treatment in Vibra Cell sonicator (Sonics & Material INC.). The lysates were centrifuged at 5.000 *g* for 20 min at 4 °C, and recovered supernatants were stored at −80 °C. The concentration of protein in *Giardia* extracts was determined using the bicinchoninic acid assay (BCA)^62^.

### Raw 264.7 cells-*Giardia* interaction

Initial experiments were performed to define the ratio of Raw 264.7 and *Giardia* cells and the co-incubation time periods. *G. lamblia* cells in log-phase of growth were washed twice in PBS, counted in a Neubauer cell-counter chamber before dilution in complete DMEM, and added to the macrophage-like cells culture at indicated number/ratios.

For the interaction assays, Raw 264.7 cells (6 × 10^5^ cells/well/ml) were culture in 24-well microplates in growth medium at 37 °C for 14 h. Following this period, macrophage-like cells were either maintained in culture medium (control), or incubated with *G. lamblia* trophozoites (3 × 10^6^ parasites/well) or *G. lamblia* extract (20 µg protein) for one hour. Later, LPS (1 μg/ml) was used to activate macrophage-like cells during 30 min or 8 h depending on the experiments.

Cell lysates were obtained by disruption in RIPA buffer (50 mM Tris-HCl, pH 8.0, 1% Nonidet P-40, 150 mM NaCl, 0.5% sodium deoxycholate, 0.1% SDS and 2 mM EDTA) freshly supplemented with 1 mM DTT, protease and phosphatase inhibitor cocktails followed by sonication (three times for 4 s at 40μm peak to peak) to decrease viscosity^63^. The nuclei and the insoluble cells debris were removed by centrifugation at 12.000 *g* for 10 min at 4 °C and supernatants used as total cell lysates. Protein concentration was determined using the BCA and cells lysates were denatured at 95 °C for 10 min in sample buffer (0.125 mM Tris, pH 6.8, 2% (w/v) SDS, 100 mM DTT, 10% glycerol and bromophenol blue).

For determine whether parasite effects on macrophage-like cells occur via direct contact with *G. lamblia* or via excretory-secretory products (ESP), experiments were performed with transwell inserts. For this, macrophage-like cells (3 × 10^6^ cells/well) were culture on 6-well microplate in 5 ml medium and allowed to adhere overnight. Subsequently, membrane inserts (0.4 µM pore size) were placed in each well, and maintained in culture medium (control) or *G. lamblia* trophozoites (1.5 × 10^7^) were added on top of insert for 1 h, before addition of LPS (1 μg/ml) to macrophage-like cells.

Additionally, some wells were supplemented with several concentrations of L-arginine (final concentration 0.8 mM, 10.4 mM and 20.4 mM) to ensure that NO production by macrophage-like cells was not limited by the depletion of this amino acid during *Giardia* metabolism.

### Human macrophages-*Giardia* interaction

Human macrophages for seven day cultures were maintained in medium (control cells), or exposure to *G. lamblia* trophozoites (3 × 10^6^ parasites/well) during one hour at 37 °C. Later, human macrophage cells were activated with LPS (1 μg/ml) during 30 min or 8 h depending on the experiments. The macrophage cells lysates were obtained as described for Raw 264.7 cells.

### Protein quantification

Protein concentration of macrophage cells lysates and *G. lamblia* extracts was estimated by the BCA assay, according to manufacturer’s instructions, employing bovine serum albumin (BSA) as standard^62^. Each sample was assayed in triplicate and blanks were included in all assays.

### Nitrite production

A colorimetric reaction with the Griess reagent was used to determine the concentration of nitric oxide (NO) by the accumulation of nitrite in the culture supernatants^64^. In brief, supernatants from co-culture of Raw 264.7 cells and G*iardia* were centrifuged at 800 × *g* for 5 min to remove the parasites. Supernatants were then diluted with equal volumes of Griess reagent [0.1% (w/v) N-(1-naphthyl)-ethylenediamine dihydrochloride and 1% (w/v) sulphanilamide containing 5% (w/v) H_3_PO_4_] and incubated at room temperature during 30 min, in the dark. The absorbance at 530 nm was measured in an automated microplate reader (Synergy™ HT, BioTek) and nitrite concentration was determined from a regression analysis using serial dilutions of sodium nitrite as standard.

### Effect of *Giardia* extracts on macrophage inflammatory proteins

Macrophage-like cells lysates (40 µg) and *G. lamblia* extracts (30 µg, 20 µg, 10 µg, 5 µg, 2.5 µg and 1 µg) were thawed and incubated together 1 h at room temperature. Following this period, cell lysates were denatured at 95 °C, for 10 min, in sample buffer (0.125 mM Tris pH 6.8; 2%, w/v SDS; 100 mM DTT; 10% glycerol and bromophenol blue) for its use in Western blot analysis. Western blot analysis was performed to evaluate the level of COX-2, iNOS and NF-κB p65^RelA^^63^.

In order to determine if *G. lamblia* proteases are involved on proteolytic cleavage of studied proteins, protease inhibitors were tested in *G. lamblia* and macrophage-like cells interaction assays^65^. EDTA (5 mM), PHEN (10 mM), E-64 (10 μM), LEUP (50 µM), PEPA (1 μM), PEPAP (1 µM), PMSF (1 mM), and AEBSF (1 mM) were incubated with *G. lamblia* extracts (10 µg) at room temperature during 30 min. Then 30 µg of macrophage-like cells lysate were added and incubated for 1 h at room temperature. Following this period, denaturing buffer was added and samples denatured 10 min at 95 °C. Subsequently, western blot analysis was performed.

### Western blot analysis

Western blot analysis was performed to evaluate the effects of *G. lamblia* (trophozoites and extracts) on the activation of MAPKs and on the expression of iNOS and COX-2 proteins.

Proteins were eletrophoretically separated as described previously^49^. Then blots were incubated overnight at 4 °C with the primary antibodies against the different proteins to be studied as follow: COX-2 (1:10.000), iNOS (1:7500), phospho-p38 MAPK (1:1000), phospho-JNK (1:1000), and NF-κB p65^RelA^ (1:1000). After washing three times with TBS-T, membranes were incubated for 2 h at room temperature with alkaline phosphatase-conjugated anti-rabbit or anti-mouse antibodies (1:20.000). Protein detection was performed using enhanced chemifluorescence system and the membranes were scanned for blue excited fluorescence on the Typhoon imager (GE Healthcare). The generated signals were analyzed using software TotalLab TL120 (Nonlinear Dynamics). Equivalent protein loading was verified by stripping the membranes and reprobing with anti-tubulin antibody.

### Substrate-gel electrophoresis

Proteinase activities in *Giardia* extracts were examined by zymographic assays. The *Giardia* extracts were analysed in 10% SDS-PAGE gels, containing gelatin (0.1%) and BSA (0.1%) as protein substrates^65^. *Giardia* extracts (20 µg, 10 µg, 5 µg and 1 µg) were solubilized in zymography sample buffer and loaded on gelatin or BSA copolymerized gels, respectively. Following electrophoresis, gels were renatured by washing twice for 30 min with 2.5% Triton X-100, and washed with distilled water before incubating for 16 h at 37 °C in digestion buffer (0.1 M phosphate buffer pH 7.4, 5 mM Ca^2+^). Gels were stained with 0.25% Coomassie Blue R250 in 50% methanol and 10% acetic acid and destaining with 25% methanol and 5% acetic acid solution. Proteolysis was visualized as clear bands against a blue background.

In order to characterize the proteolytic activity, *Giardia* extracts (10 µg) were pre-incubated with each one of the protease inhibitors EDTA (5 mM), PHEN (10 mM), E-64 (10 μM), LEUP (50 µM), PEPA (1 μM), PEPAP (1 µM), PMSF (1 mM), and AEBSF (1 mM) and loaded on gels. After electrophoresis and Triton X-100 treatment, the gels were incubated in the digestion buffer supplemented with each inhibitor and the assay was conducted as described above.

### Analysis of gene transcription by quantitative reverse transcription PCR (RT-qPCR)

For assessment of gene transcription during macrophage-*Giardia* interaction, Raw 264.7 cells (1.5 × 10^6^/well) were plated in 24-well microplates in 1.2 ml culture medium and incubated at 37 °C in a humidified atmosphere of 95% air and 5% CO^2^ for 14 h. Subsequently, 7.5 × 10^6^ parasites were added to each well and the samples for RNA extraction were taken after 6 h of co-infection. Therefore, trophozoites forms were detached by chilling the microplate on ice for 20 min, supernatant was removed, and macrophage cells were washed several times in cold PBS. Total RNA was isolated from macrophage cells with RNAzol reagent, according to the manufacturer’s instructions, and the concentration was spectrophotometric determined by measurement of OD260 (NanoDrop,Thermo Scientific). RNA samples were stored in *Storage Solution* at −80 °C until they were used. Briefly, 1 μg of RNA was reverse-transcribed using iScript Select cDNA Synthesis kit, and qPCR were performed, in duplicate for each biological sample, on Bio-Rad My Cycler iQ5, using Sso Fast Eva Green Supermix. The results were normalized using *Gapdh* as a reference gene. The sequences of the primers *Tnfa*, *Il1b*, *Il6*, *Il10*, *Ccl3* and *Ccl4* (Supplementary Table 1) were described in previous works of our lab^63^.

### Calculation of qPCR results

Gene expression changes were calculated by the Pfaffl method, a variation of detla/delta CT method corrected for gene-specific efficiencies, and to report gene expression changes as relative fold changes compared to control samples^66^. Obtained fold changes were then processed as previously described^63^. Briefly, as the qPCR results are presented as ratios of treated samples to untreated cells (control), the distribution of data does not follow a normal distribution. Therefore, a two-base logarithmic transformation was used to make observations symmetric and closer to a normal distribution. If × represents the fold change of the gene in one sample, then the two-base logarithmic transformation [log2(×)] is ln(×)/ln(2). Therefore, fold changes of 2 and 0.5 correspond to mean log2 values of 1 and −1, respectively.

### Immunocytochemistry analysis

The effect of *G. lamblia* trophozoites on the levels and localization of the NF-κB p65^RelA^ protein was also evaluated by immunocytochemistry analysis. Raw 264.7 cells (5 × 10^4^cells/well) were cultured in µ-slides 8 wells (IBIDI) and stimulated as described above. After indicated periods, cells were washed with ice-cold PBS, fixed with 4% paraformaldehyde in PBS for 15 min with 0.3% Triton X-100. The macrophage cells were then blocked for 1 h at room temperature with 10% goat serum and incubated overnight at 4 °C with a rabbit polyclonal anti-p65 antibody (1:100). After washing with PBS, macrophage cells were incubated, for 1 h at room temperature, with Alexa 488-conjugated goat anti-rabbit antibody (1:400) and Alexa Flúor 555 Phalloidin (1:200). Finally, mounting medium containing DAPI was added to the wells and cells visualized using an Axio Observer.Z1 inverted microscope (Zeiss). In each experiment, the optimal acquisition parameters were defined for the control cells and then maintained for all the other conditions within the same experiment. Images presented in the Results section are representative images of three separate experiments.

### Statistical analysis

The results of Western Blot are expressed as mean ± SEM from at least three independent experiments and analyzed by one-way analysis of variance (ANOVA), followed by Dunnett´s test, using version 6.0d of GraphPad Prism (GraphPad Software, San Diego, CA, USA). The data of qPCR are presented as mean ± SEM, and the means were statistically compared using the one-way ANOVA test, followed by Bonferroni´s multiple comparison post-test. The significance level was ^*^p < 0.05, ^**^p < 0.01 and ^***^p < 0.001, when compared to control; ^#^p < 0.05, ^##^p < 0.01 and ^###^p < 0.001, ^####^p < 0.0001 when compared to LPS.

## Supplementary information

Supplementary Information.
