# Supplementary material for: Giardia lamblia Decreases NF-κB p65RelA Protein Levels and Modulates LPS-Induced Pro-Inflammatory Response in Macrophages
Source: Sci Rep. 2020 Apr 10;10:6234. doi: 10.1038/s41598-020-63231-0 (PMC7148380; doi:10.1038/s41598-020-63231-0)
Supplement: Supplementary file 1 — Supplementary Information. [file 41598_2020_63231_MOESM1_ESM.pdf]

Supplementary Information

Giardia lamblia Decreases NF-κB p65^{RelA} Protein Levels and Modulates LPS-Induced Pro-Inflammatory Response in Macrophages

Clarissa Perez Faria[1,2], Bruno Miguel Neves [3], Ágata Lourenço[1,2], Maria Teresa Cruz [1,2], João D Martins [2], Ana Silva [2], Sónia Pereira [1,2], Maria Céu Sousa[1,2*]

[1]Faculty of Pharmacy, University of Coimbra, Coimbra, Portugal;
[2]Center for Neuroscience and Cell Biology, University of Coimbra, Coimbra, Portugal;
[3]Department of Medical Sciences and Institute of Biomedicine – iBiMED, University of Aveiro, 3810-193 Aveiro, Portugal

Supplementary Table 1: Primer sequences for targeted cDNAs

**Primer**	**5´-3´ sequence F: forward; R: Reverse**	**RefSeq ID**
Gapdh	F: GCCTTCCGTGTTCCTACC<br>R: GCCTGCTTCACCACCTTC	NM_008084
Il1b	F: ACCTGTCCTGTGTAATGAAAG<br>R: GCTTGTGCTCTGCTTGTG	NM_008361
Il6	F: TTCCATCCAGTTGCCTTC<br>R: TTCTCATTTCCACGATTTCC	NM_031168
Il10	F: CCCTTTGCTATGGTGTCCTTTC<br>R: ATCTCCCTGGTTTCTCTTCCC	NM_010548
Tnf-α	F: CAAGGGACTAGCCAGGAG<br>R: TGCCTCTTCTGCCAGTTC	NM_013693
Ccl3	F: AGCCAGGTGTCATTTTCC<br>R: CATTCAGTTCCAGGTCAGT	NM_011337
Ccl4	F: TCACTGAGAAGCGTCCTT<br>R: GTCATATCCACAATAGCAGAGAA	NM_013652

Supplementary Fig. S1 Effects of *Giardia* trophozoites extracts on nitrite production in macrophages after supplementation with several concentrations of arginine.

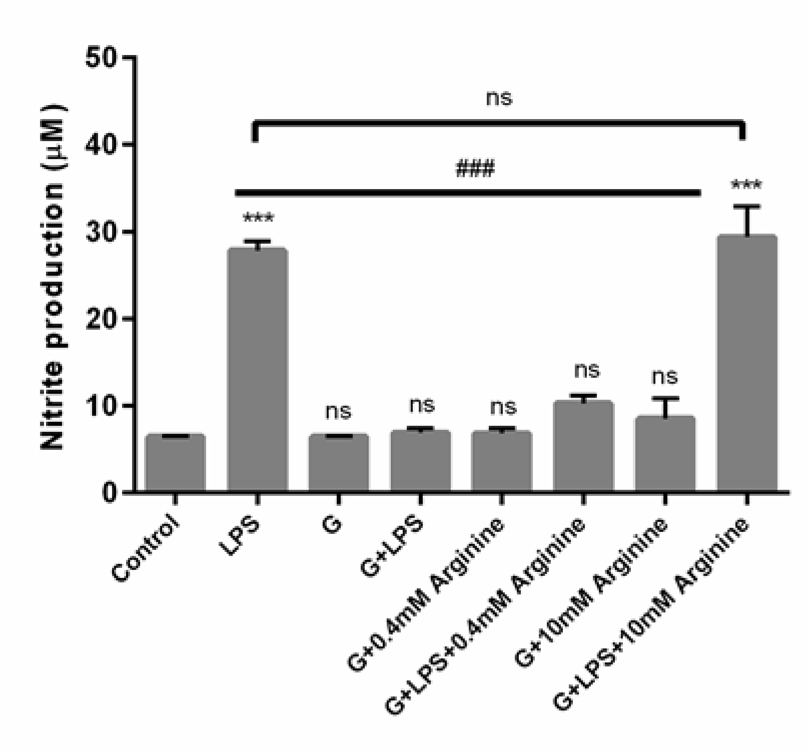

Raw 264.7 cells (6 x 10^5 cells) were maintained in culture medium (control), or pre-incubated with *G. lamblia* extract (20µg) for 1h with DMEM culture medium supplemented with 0.4mM, 10mM or 20mM of L-arginine (final concentration 0.8mM, 10.4mM and 20.4mM), and then activated with 1µg/ml LPS for 8h. Each value represents the mean ± SEM from at least 3 independent experiments ($^{***}p<0.001$, control vs treatment; $^{\#\#\#}p<0.001$, LPS vs treatment; ns, not significant).

Supplementary Fig. S2 Effect of *Giardia lamblia* on the total levels of COX-2 expression in human macrophage.

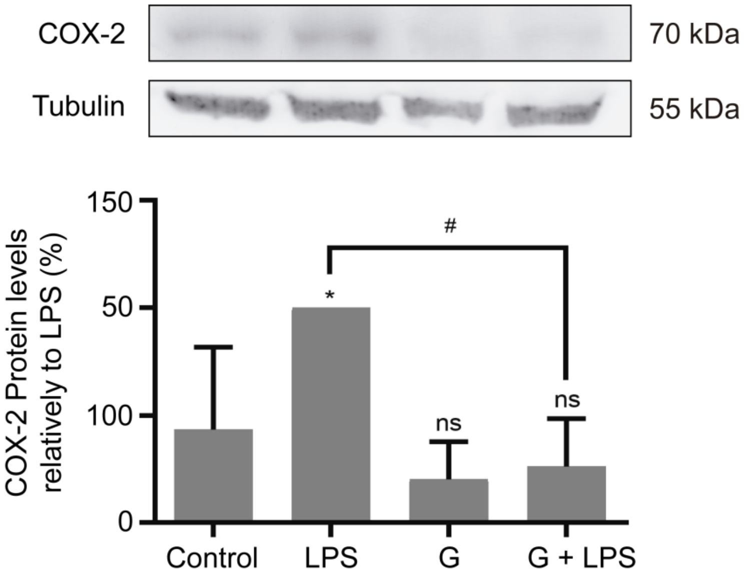

Effects of *G. lamblia* trophozoites on LPS-induced COX-2 protein expression in human macrophage. Macrophage (6 x 10^5 cells) were maintained in culture medium (control), or pre-incubated with *G. lamblia* (3 x 10^6 cells) for 1h, and then activated with 1μg/ml LPS for 8h. COX-2 expression was analyzed by Western blot using a specific anti-COX-2 antibody and anti-tubulin antibody was used to confirm equal protein loading. The blot shown is representative of 3 blots yielding similar results. Results were expressed as percentage of COX-2 protein levels relatively to LPS. Each value represents the mean ± SEM from at least 3 independent experiments ($^*p<0.05$, compared to control; $^\#p<0.05$, LPS vs treatment; ns, not significant).

Supplementary Fig. S3 Macrophages viability.

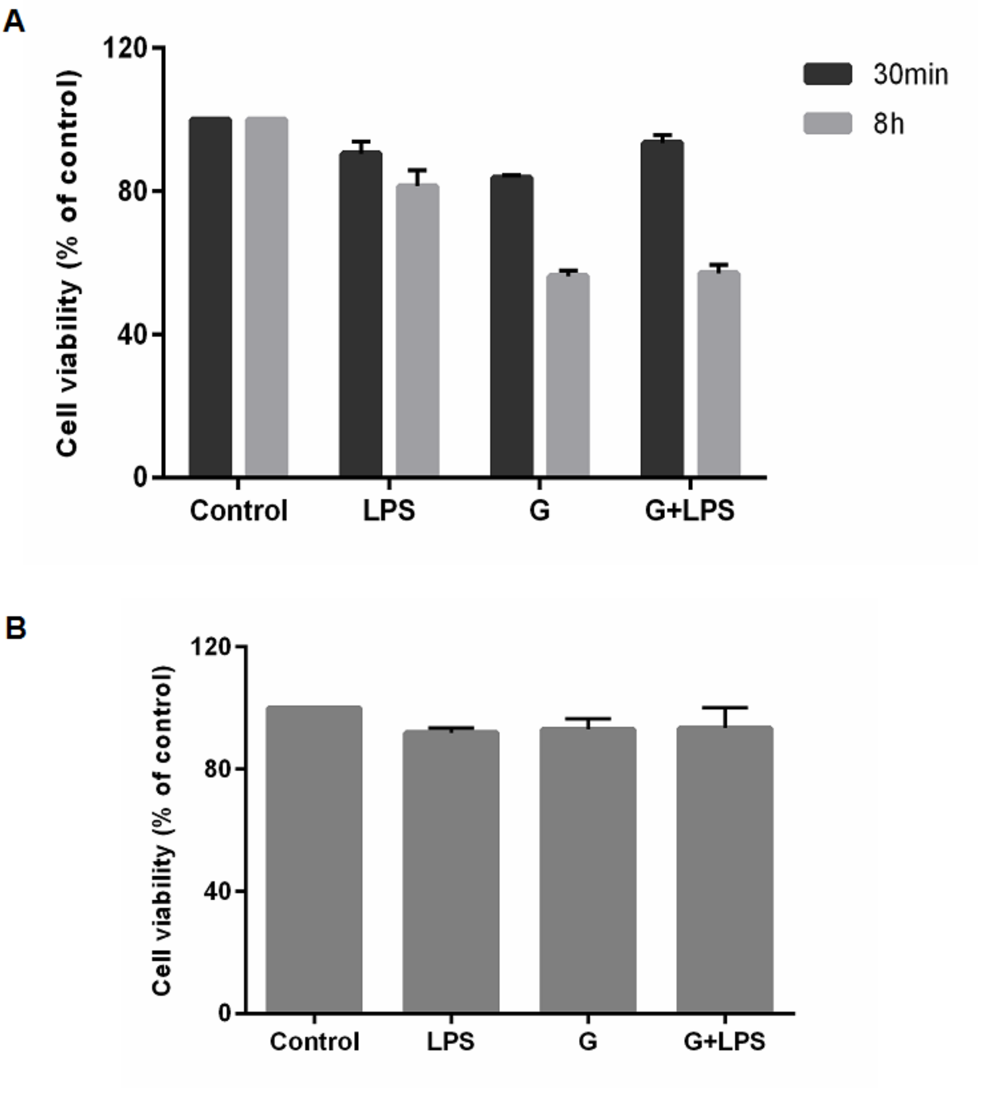

Evaluation of macrophage viability in the presence or in the absence of *Giardia lamblia* and/or LPS (1µg/ml). (A) Cells exposed to *Giardia lamblia* trophozoites for 30 minutes and 8 hours or (B) to *Giardia lamblia* extracts for 8 hours.

Supplementary Fig. S4 Effect of *Giardia lamblia* extracts on the total levels of NF-κB p65, iNOS and COX-2.

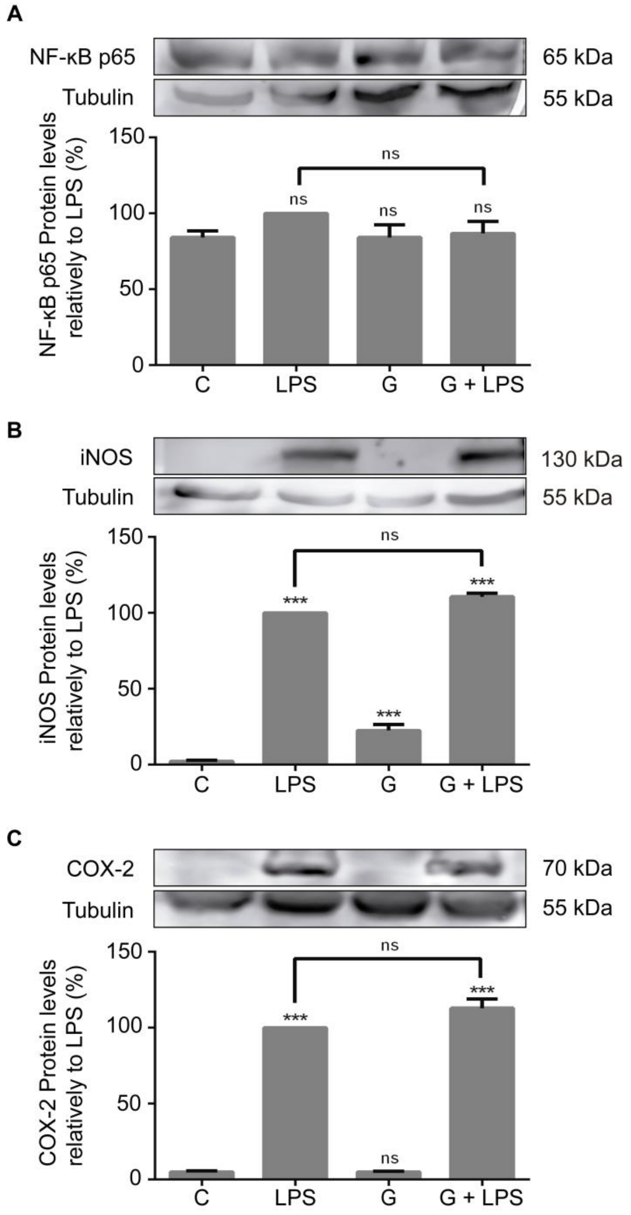

Raw 264.7 cells (6 x 10^5 cells) were maintained in the culture medium (control), or pre-incubated with *G. lamblia* extract (20µg) for 1 hour, and then activated with 1µg/ml LPS for 30min (NF-κB p65) or 8h (iNOS and COX-2). Total cell extracts were analyzed by Western blot using antibodies against (A) NF-κB $p65^{RelA}$, (B) iNOS and (C) COX-2. An anti-tubulin antibody was used to confirm equal protein loading. The blot shown is representative of 3 blots yielding similar results. Each value represents the mean ± SEM from at least 3 independent experiments ($^{***}p<0.001$ compared to control; ns, not significant).

Supplementary Fig. S5 Gelatin-zymography analysis of *Giardia lamblia* proteolytic activity.

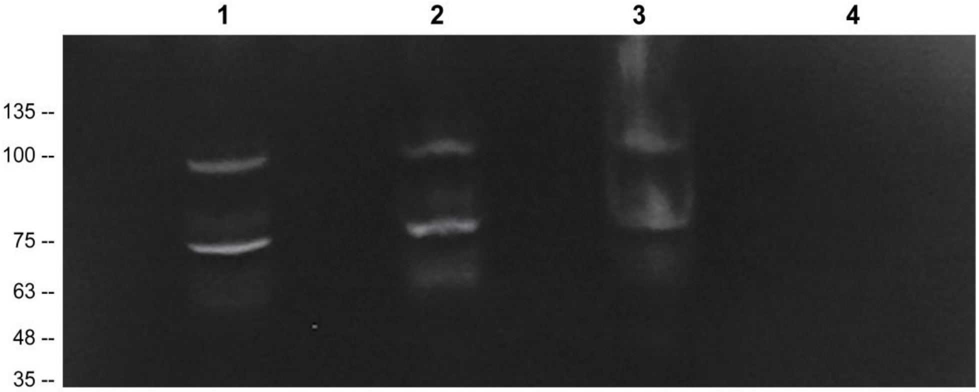

Giardia extracts were loaded in the gel at different protein concentrations: 20µg (lane 1), 10µg (lane 2), 5µg (lane 3) and 1µg (lane 4). Molecular mass markers are shown on the left in kilodaltons (kDa).

Supplementary Fig. S6 Full-length blots of Figure 2.

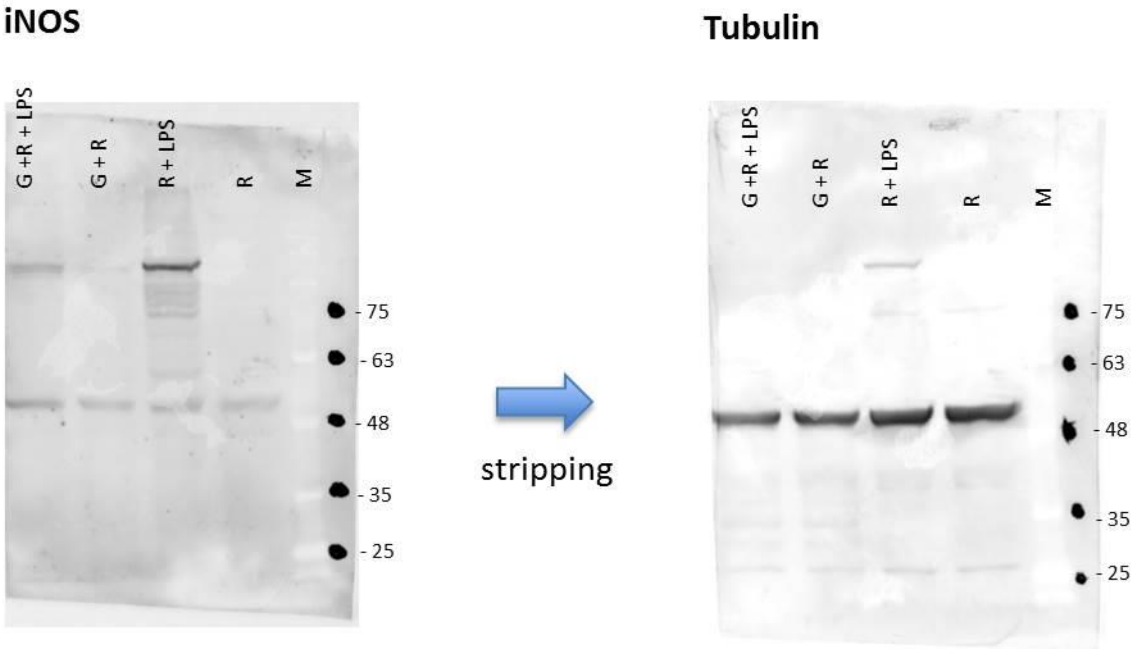

Effects of *Giardia* trophozoites on LPS-induced iNOS protein expression and nitrite production in macrophages.

Supplementary Fig. S7 Full-length blots of Figure 4.

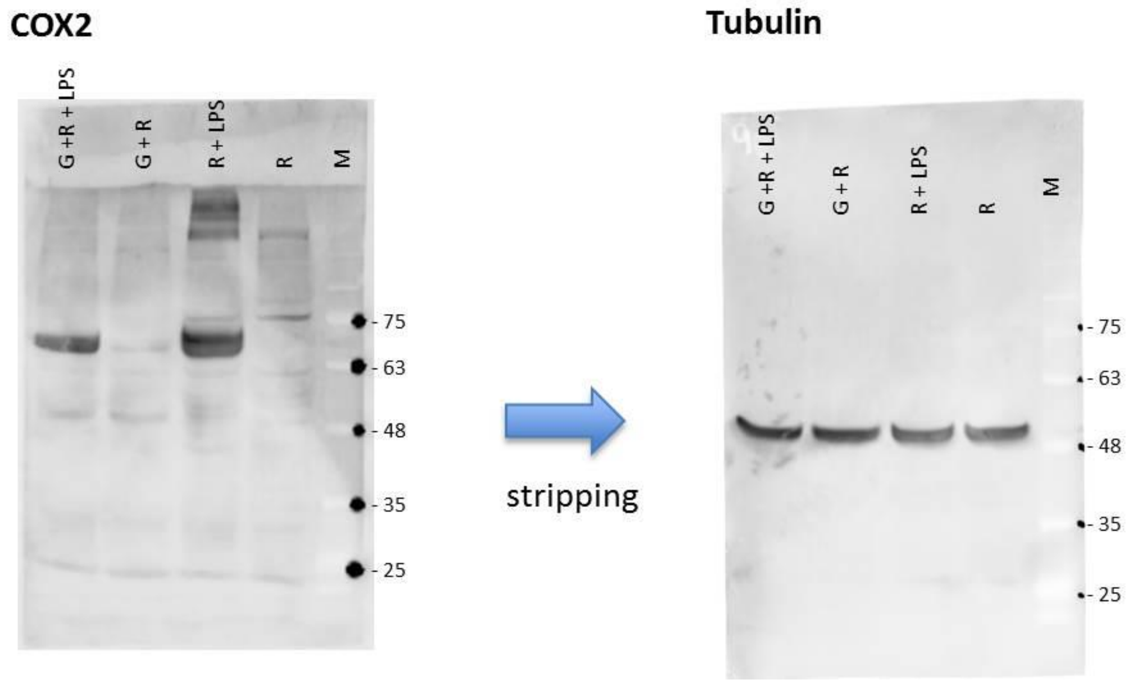

Effects of *G. lamblia* trophozoites on LPS-induced COX-2 protein expression in macrophages.

Supplementary Fig. S8 Full-length blots of Figure 5.

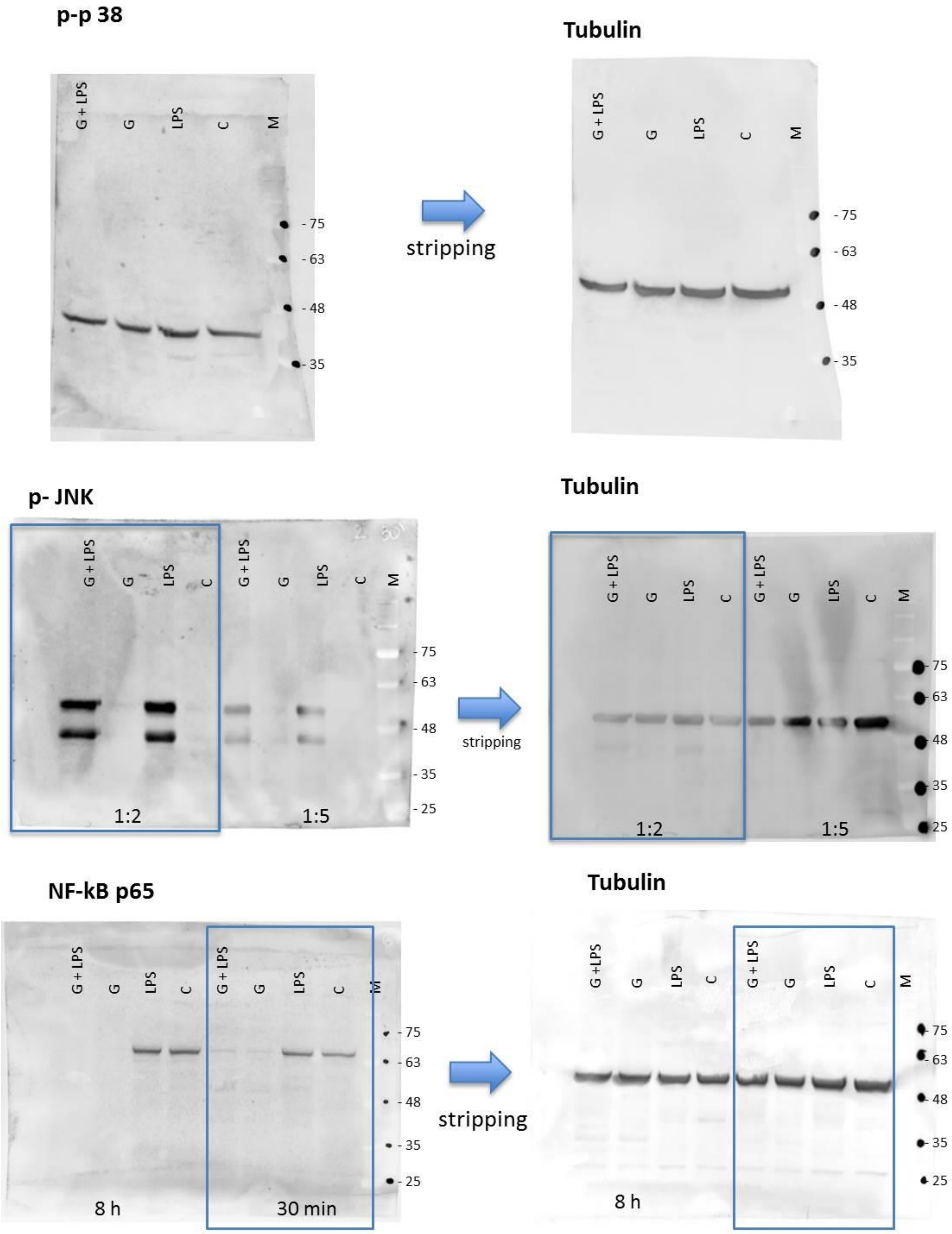

Effects of trophozoites on NF-κB and MAPKs signaling pathways.

The samples used in the main figure are surrounded by a blue line.

Supplementary Fig. S9 Full-length blots of Figure 8.

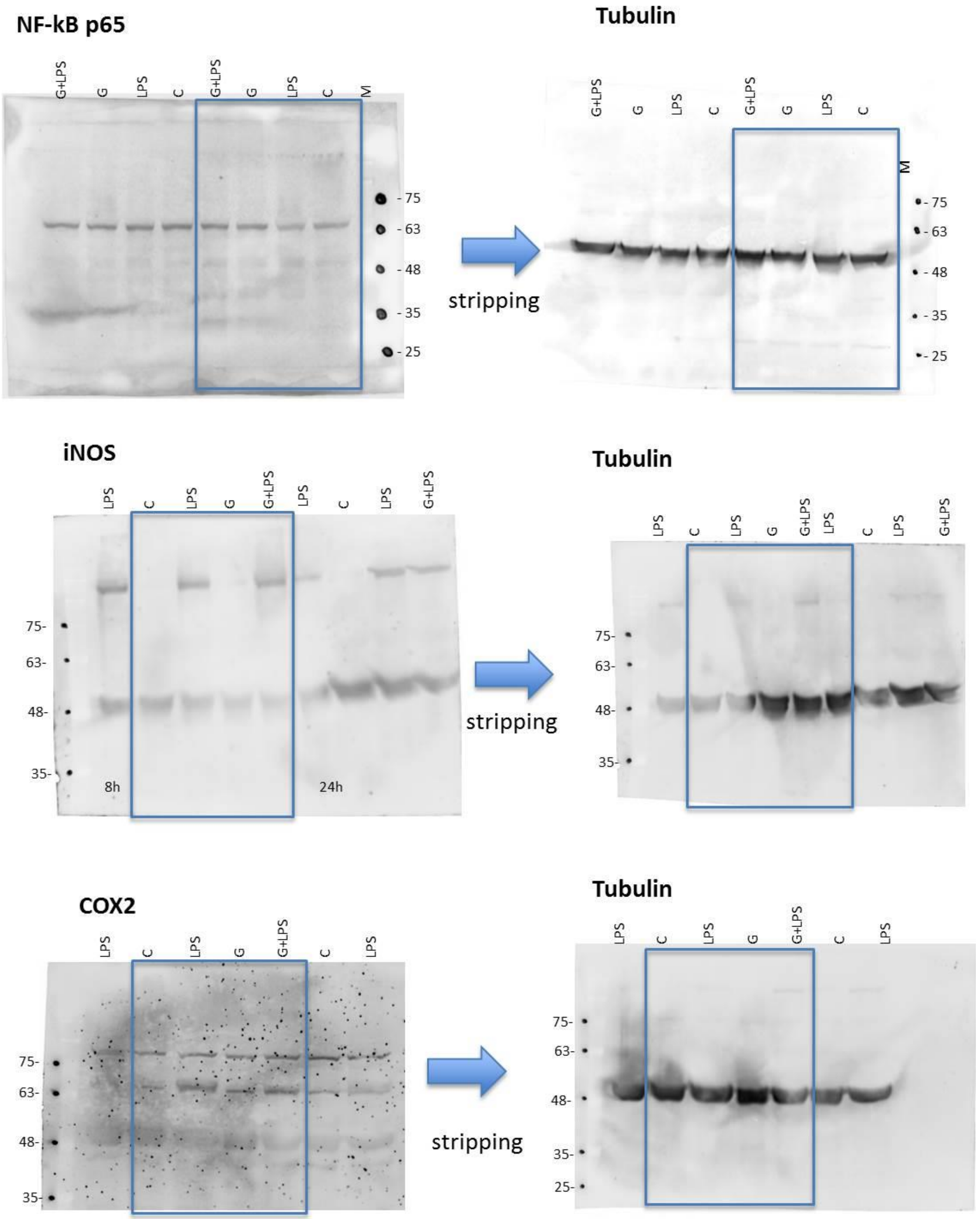

Effects of trophozoites excretory-secretory products on the total levels of NF-κB p65, iNOS and COX-2.

The samples used in the main figure are surrounded by a blue line.

Supplementary Fig. S10 Full-length blots of Figure 9.

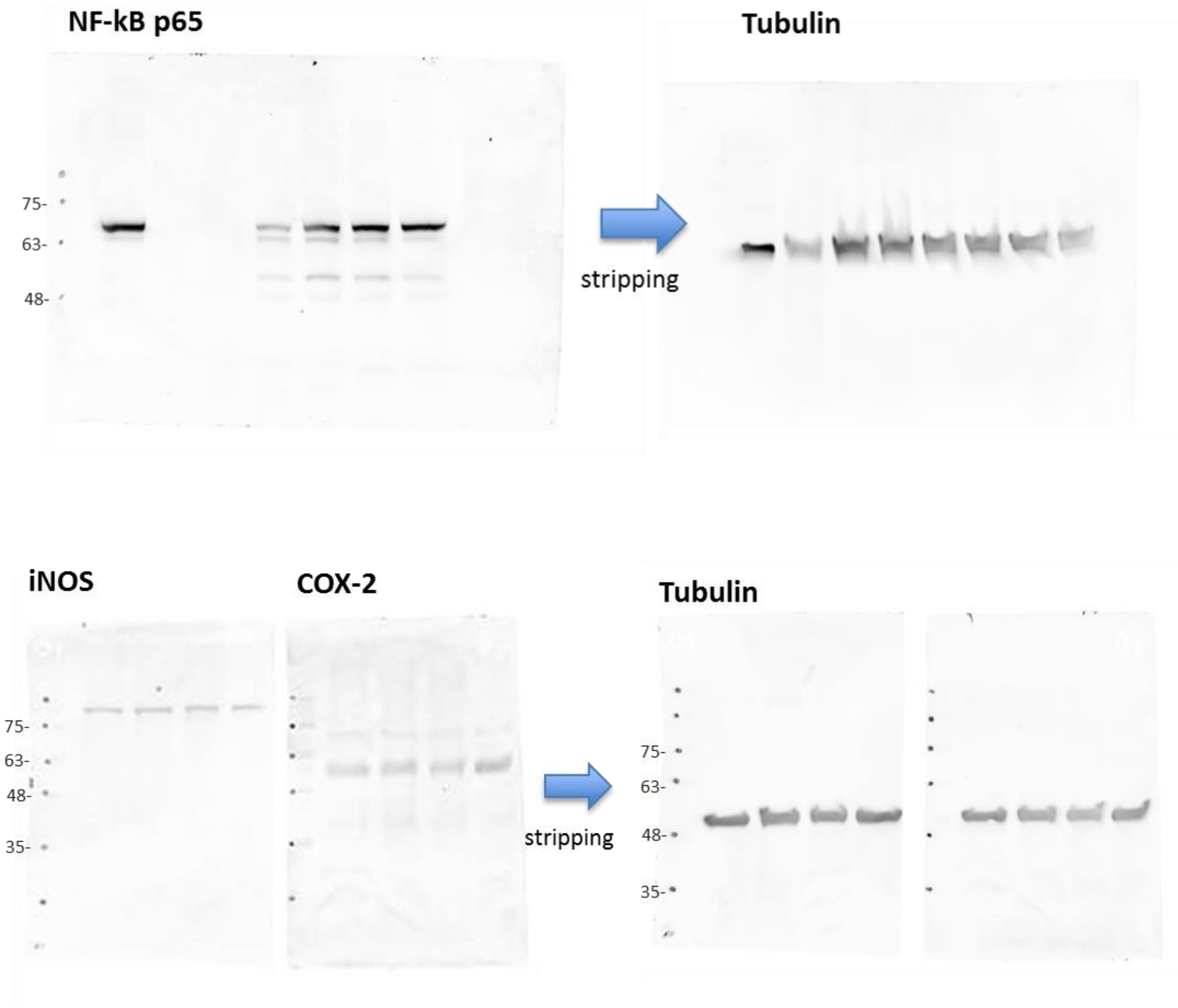

Proteolytic activity of *Giardia lamblia* extracts over NF-κB p65^{RelA}, iNOS and COX-2.

Supplementary Fig. S11 Full-length blots of Figure 10.

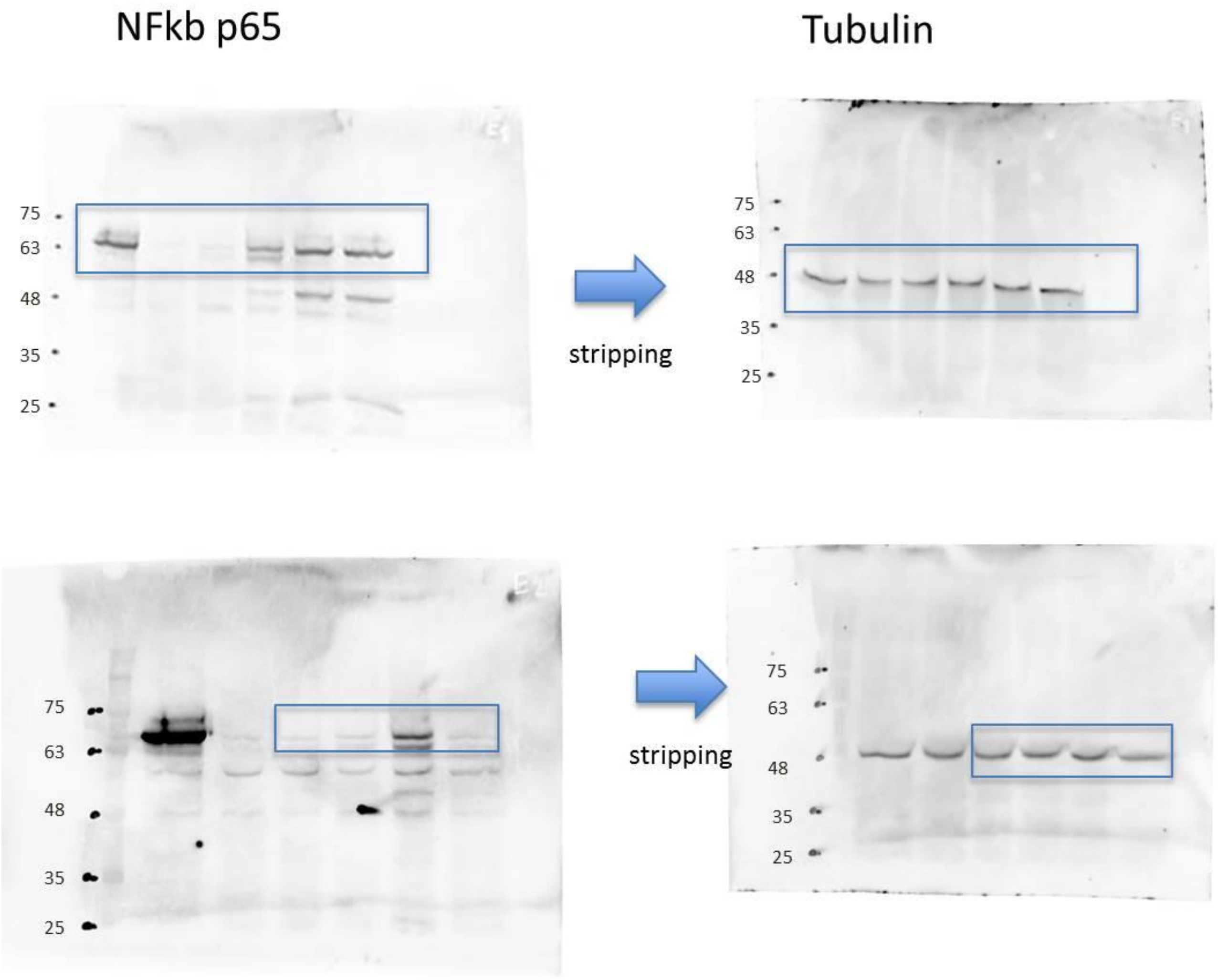

Effects of protease inhibitors on *Giardia lamblia* protease activities and *Giardia*-promoted NF-κB p65 protein degradation.

The samples used in the main figure are surrounded by a blue line.
